# Single gold-bridged nanoprobes for identification of single point DNA mutations

**DOI:** 10.1038/s41467-019-08769-y

**Published:** 2019-02-19

**Authors:** Xingyi Ma, Sojin Song, Soohyun Kim, Mi-sun Kwon, Hyunsook Lee, Wounjhang Park, Sang Jun Sim

**Affiliations:** 1 Department of Chemical & Biological Engineering, Korea University, Seoul, 136713 Republic of Korea; 2 Department of Biological Sciences & Institute of Molecular Biology and Genetics (IMBG), Seoul National University, Seoul, 151742 Republic of Korea; 3 Department of Electrical, Computer & Energy Engineering, Materials Science & Engineering Program, University of Colorado, Boulder, CO 80309 USA

## Abstract

Consensus ranking of protein affinity to identify point mutations has not been established. Therefore, analytical techniques that can detect subtle variations without interfering with native biomolecular interactions are required. Here we report a rapid method to identify point mutations by a single nanoparticle sensing system. DNA-directed gold crystallization forms rod-like nanoparticles with bridges based on structural design. The nanoparticles enhance Rayleigh light scattering, achieving high refractive-index sensitivity, and enable the system to monitor even a small number of protein-DNA binding events without interference. Analysis of the binding affinity can compile an atlas to distinguish the potential of various point mutations recognized by MutS protein. We use the atlas to analyze the presence and type of single point mutations in *BRCA1* from samples of human breast and ovarian cancer cell lines. The strategy of synthesis-by-design of plasmonic nanoparticles for sensors enables direct identification of subtle biomolecular binding distortions and genetic alterations.

## Abstract (web summary)

The identification of single point mutations is an important step toward personalizing detection and treatment. Here the authors design a gold-bridged nanoparticle for sensing MutS affinities to point mutations, and compile an atlas of the affinity data for detecting *BRCA1* mutations in cell lines.

## Introduction

Many diseases have a genetic component; their detections therefore require a clear understanding of underlying mutations^1^. For example, approximately 12% of women will develop breast cancer during their lives, with the highest risk conferred by *BRCA1* mutations (59–87%)^2^. Most methods for identifying gene mutations rely on sequencing^3^, but a method that can detect the presence and identity of single mutant bases without prior knowledge of the sequence—ideally without artifacts from labels and the in vitro environment—is desired. The specificity of the biological interaction between the post-replicative mismatch repair (MMR) initiation protein MutS and mismatched DNA enables detection of nucleotide polymorphisms by methods such as single-molecule fluorescence resonance energy transfer (smFRET); however, these require labor-intensive steps such as labeling of MutS or fabrication of radioactive probes for DNA^4–7^. Visualizing MutS molecules by atomic force microscopy is complicated and difficult to apply to biomedical sensors^8,9^. On the other hand, bulk measurements of point mutations by gel mobility shift and filter/chip binding assays do not output real-time information on molecular interactions^10–13^, whereas measurements by surface plasmon resonance (SPR), electrochemical assay, and quartz crystal microbalance (QCM) are time consuming and inefficient (detailed in Supplementary Note 1)^14–16^. Accurate methods for quantifying protein binding affinity over arbitrarily full DNA footprints are currently limited to computational approaches^17^. An atlas of nucleic acid–MutS binding affinities at single-base resolution that reveals changes in gene regulation in disease states and describes the relationship between point mutation type and repair efficiency of the MMR system would be highly useful, but is currently lacking^18^.

For advanced sensor applications, plasmonic nanoparticles (NPs) have attracted interest due to their ability to interact with light and produce localized SPR (LSPR). The collective oscillation of electrons in the nanostructure at a given resonant frequency transduces changes in the local refractive index (RI) into shifts in the plasmonic bands of their absorption and scattering spectra^19^. The sensing scale can be reduced to a single NP; such single NP sensing (sNPS) can relay local biological information on a nanometer scale in which the limit of detection (LOD) reaches countable numbers of molecules using a very small sensing volume. For example, the MutS protein is 125 × 90 × 55 Å^20^; thus, adsorption of MutS onto a single NP can drastically alter the collective oscillatory behavior of its surface electrons, resulting in wavelength shifts in the NP spectra^21^. In contrast, most other sensing techniques using bulk solutions or planar surfaces show a limited ability to localize and separate sensing elements and are limited by slow molecular diffusion, stochastic binding, and frequent dissociation of complexed biomolecules with consequent disequilibrium of reactions, resulting in signal fluctuations with a low signal-to-noise ratio (S/N). An sNPS sensor is a tiny probe capable of high-throughput and parallel readout in which the structure and localized sensing volume/area of the NP are essential for RI sensitivity. Studies have shown that rod-like NPs exhibit the highest sensitivity to changes in RI^22^. Nanogap and nanobridge structures are associated with and generate strong optical signals by plasmonic coupling, further enhancing the local field to generate distinct spectral responses^23,24^. However, synthesizing colloidal plasmonic NPs with a predefined structure is challenging due to the difficulty in manipulating atoms that are transient in solution^25^. Moreover, chemically synthesized nanostructures are restricted to a highly symmetric shape with identical surface facets (e.g., nanospheres (NSphs), nanorods (NRs), nanocubes, nanodisks, and others)^26^. Two research groups recently achieved breakthroughs in synthesis-by-design at sub-5 nm precision using a programmable biomolecule—i.e., DNA—to create nanoplasmonic particles either by casting in DNA molds^27^ or using DNA frameworks^28^. Structurally programmable NPs could overcome sNPS limitations such as low sensitivity and reproducibility.

Here we report the synthesis-by-design of plasmonic nanostructures for detecting a countable number of biomolecule binding events. The nanostructure with a rod shape and one nanobridge (bridged NP) show higher RI sensitivity than similarly sized NSphs, NRs, and NPs with a nanogap (NPs-gap). The bridged NPs are synthesized in solution by highly controlled direction-specific crystallization whereby double-stranded DNA (dsDNA) molecules with adjustable lengths and surface charges subtly regulate Au atom crystallization, a process distinct from Au metallization. The Au-bridged NPs utilize low-energy white light as a signal source in an sNPS system, which preserve intrinsic MutS–DNA interactions and enable different point mutations to be recognized by MutS with high speed, resolution, and fidelity. The method generates the atlas of MutS affinities to various synthetic DNA samples, and further applies to analyze genomic DNA extracted from human breast and ovarian cancer cells for directly identifying mutated single bases.

## Results

### NP design with numerical simulations

**Fig. 1:**
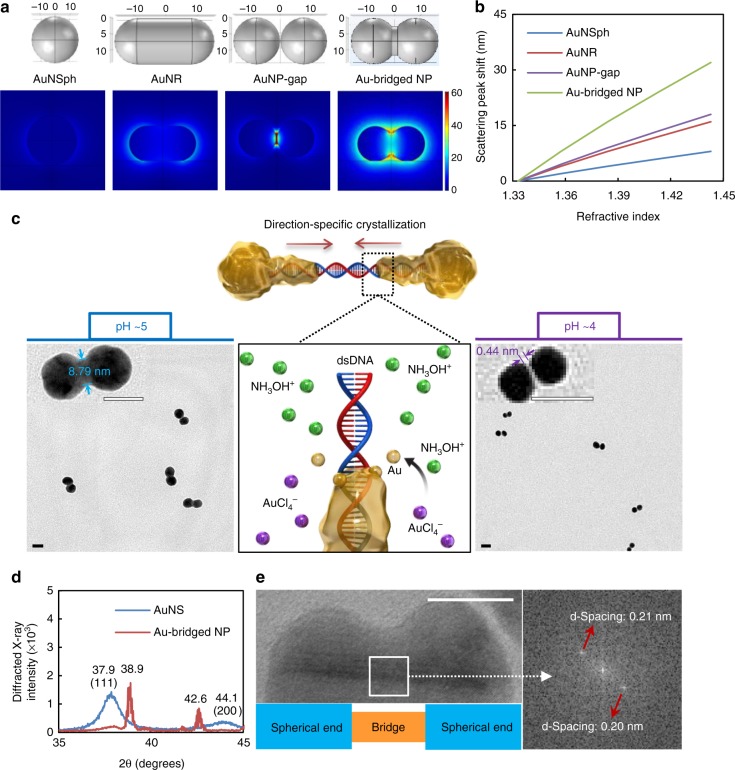
Synthesis-by-design of plasmonic nanoparticles (NPs) in solution. **a** Illustrations of the designed NP models (upper; dimensional unit, nm) and plasmon resonance electric field patterns (below; unit, V m^−1^) generated by numerical simulations. **b** Linear fits to localized surface plasmon resonance (LSPR) wavelength shifts vs. changes in the refractive index (RI) of the NP surroundings. **c** Schematic diagram showing the early stage of direction-specific, contour-following, and shape-controlled crystallization of Au atoms by reducing AuCl_4_^−^ with NH_3_OH^+^. The water interface of DNA provides precise controllability under the synthetic conditions at pH 5 and 4, generating NPs with nanobridges and nanogaps respectively, as shown in the transmission electron microscopy (TEM) images. Scale bars, 20 nm. **d** X-ray diffraction spectra of AuNSs and Au-bridged NPs. **e** High-resolution transmission electron microscopy (HR-TEM) image of the DNA-directed nanocrystal and fast Fourier transform pattern (right) of the selected area. Scale bar, 10 nm. Source data of Fig. 1b and d are provided as a Source Data file

Since each NP functions as a signal transducer in the sNPS platform, NP structure and shape should be homogeneous and controllable^19^. This excludes irregularly shaped nanocrystals (e.g., branched nanostars), since their formation is empirical rather than based on the principles of synthesis^26–28^. Furthermore, the controllability of polyhedral nanostructures is limited by the lack of chemicals that can specifically tune targeted crystal facets and thus produce NPs with a relatively high yield^29^. We therefore selected nanostructures in the shape of spheres and rods as substrates for sNPS, since both can be synthesized in a uniform and scalable manner. We also introduced structures consisting of nanogaps and nanobridges that induce distinct spectral responses and influence the magnitude of plasmonic coupling, polarization direction, signal intensity, and RI sensitivity (Fig. 1a)^23,24^. Instead of using complex biomarkers to quantify RI sensitivity, we performed optical simulations of single NPs with predesigned structures in which the RI of the surrounding medium was set to change (Supplementary Fig. 1). RI sensitivity was quantified by analyzing changes in LSPR wavelength (λ_max_) of single Au-NPs induced by different RI solutions^22^. Changes in λ_max_ corresponding to each change in RI were expressed as a linear fit (Fig. 1b) in which the slope of the line represents RI sensitivity of the NPs. Interestingly, NP-gap and bridged NP showed better performance than the NR, which was previously thought to be the optimal structure^22^. This is a direct consequence of the high field concentration provided by nanogap and nanobridge structures. In particular, the Au-bridged NP showed twofold higher sensitivity than did the AuNR, making it an ultrasensitive candidate material for sNPS fabrication.

### Synthesis-by-design of NPs

We explored the feasibility of direction-specific crystallization by which one dsDNA anchored between two Au nanoseeds (AuNSs; 5 nm in diameter) served as a directional guide for the crystallization of Au atoms into nanobridges (Supplementary Fig. 2). Interestingly, altering the surface charge of dsDNA by adjusting the pH also generated nanogaps smaller than 1 nm (Fig. 1c). At pH 5, dsDNA is slightly negatively charged and electrostatically concentrates NH_3_OH^+^. The reaction between NH_3_OH^+^ and AuCl_4_^−^ induced Au crystallization along the DNA. Consequently, Au-bridged NPs with a length of 31.15 ± 1.00 nm and a diameter of 14.38 ± 0.58 nm for the two spherical ends and 8.79 ± 0.96 nm for the bridge were formed (Supplementary Table 1). The yield of the desired morphology was 87%, and the nanostructures were in a relatively high monodispersity (Supplementary Fig. 3). In contrast, at pH 4, dsDNA is positively charged owing to its isoelectric point (pI) of 4–4.5^30^. The DNA repelled NH_3_OH^+^ by an electrostatic repulsive force, and therefore, the reaction between NH_3_OH^+^ and AuCl_4_^−^ occurred mostly near the AuNS, which further autocatalyzed the crystallization of Au atoms surrounding its surface. The reaction ended with the complete oxidation of Au ions into atoms, leaving a 0.44 nm gap between the two nanospheres (17.01 ± 1.07 nm in diameter).

Notably, crystallization occurred in specific directions from the AuNS-dsDNA boundaries to the mid-point of the dsDNA strand, with nanoscale controllability defined by the length of dsDNA. This method differs fundamentally from conventional approaches involving metallizing DNA or DNA origami, in which either sequential necklaces or continuous bulges are formed with poorly controlled structural precision (>100 nm)^31^. The directional effect of DNA in the synthesis of Au-bridged NPs was evaluated by X-ray diffraction and high-resolution transmission electron microscopy (HR-TEM) (Fig. 1d, e). The AuNSs exhibited peaks of an *fcc* structure of Au (JCPDS No. 03-0921) at 38° (111) and 44° (200). The peak positions showed clear shifts after DNA-directed crystallization, indicating that the DNA induced significant lattice strain in the Au-bridged nanostructure^26^. The narrower linewidth of the peaks indirectly reflected enlarged particle sizes. Furthermore, HR-TEM images of the nanoscale bridge regime revealed crystal planes with a spacing of 0.208 ± 0.004 nm, corresponding to (200) lattice fringes in the <100> crystallization direction^28^.

### sNPS with Au-bridged NPs

**Fig. 2:**
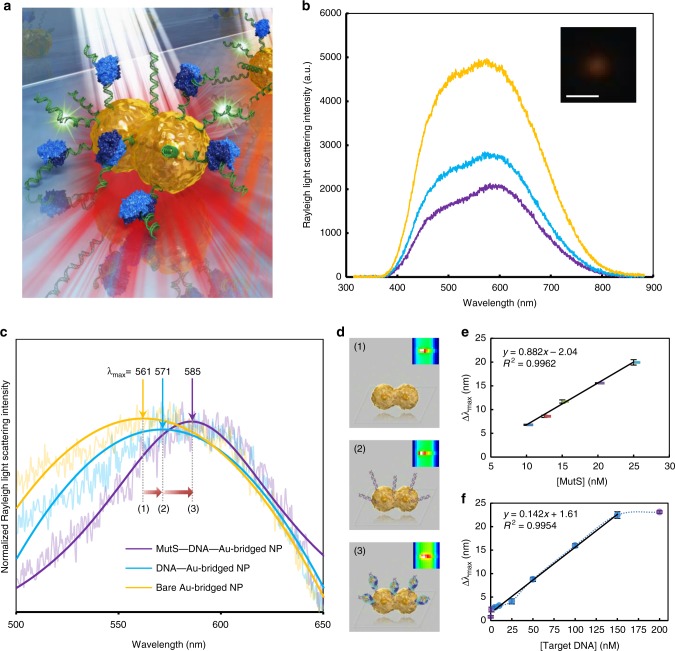
Fabrication of single nanoparticle (NP) biosensor of high sensitivity and fidelity. **a** Schematic illustration of single NP sensing (sNPS) for identifying single point DNA mutations. **b** Representative Rayleigh light scattering (RLS) spectra and in-situ dark-field microscopy image of an Au-bridged NP. Scale bar, 1 μm. **c** Localized surface plasmon resonance (LSPR) λ_max_ shifts upon binding of DNA and MutS. Same line legend for (**b**, **c**). Each step of molecular binding (1, 2, and 3) illustrated in (**d**). Insets: real-time images of a single NP obtained with charge-coupled device (CCD) camera. **e** Concentration of MutS for adequate signal response. Calibration curve showing the linear relationship between the Δλ_max_ and various concentrations of MutS. **f** Limit of detection (LOD) of the mutant DNA target. Calibration curve showing the linear relationship between the Δλ_max_ and various concentrations of target DNA. For all panels, error bars represent mean ± S.E.M. All experiments were performed in triplicate. Source data of Fig. 2b, c, e, and f are provided as a Source Data file

We investigated resonant Rayleigh light scattering (RLS) responses of a single Au-bridged NP by sNPS with a white light source (Supplementary Fig. 4). The light generated LSPR with NPs that sufficiently enhanced light scattering to allow for direct observation of individual NPs; on the other hand, the white light illumination avoided high energy and heat that could denature target biomolecules or block molecular interactions in the microfluidic reaction chamber^19^ (Fig. 2a). The RLS spectrum of a single Au-bridged NP had two surface plasmon peaks (Fig. 2b): one was related to electron oscillation in the transverse direction, resulting in a relatively weak resonance band, whereas the other was stronger and was related to electron oscillation in the longitudinal direction. We focused on longitudinal surface plasmon peaks since the longitudinal mode is more sensitive to changes in the dielectric constant of the medium than the transverse one^22^. The adsorbates of the medium were single-stranded DNA (ssDNA) and MutS protein. The probe ssDNA was anchored on Au-bridged NPs by thiol modification and then hybridized with a target ssDNA through hydrogen (H-)bonding and π–π stacking, during which process the λ_max_ red shifted from 561.1 ± 0.5 nm to 570.7 ± 0.5 nm (steps 1 and 2; Fig. 2c). MutS with a positively charged surface sequence independently contacted and caused clenching of the negatively charged DNA backbone at mismatched points with the conserved Phe-Xaa-Glu motif^32^, resulting in a red shift of up to 24 nm relative to the λ_max_ of bare NPs to 585.3 ± 0.5 nm (steps 2 and 3; Fig. 2c). According to the Mie theory, the LSPR of metallic NPs depends on the shape, size, and RI of the local dielectric environment. Using an individual NP eliminated differences in shape and size; thus, the LSPR λ_max_ shifts were attributed to changes in the NP interface upon DNA hybridization and subsequent MutS binding (Fig. 2d). To verify the specificity of the peak shifts that occurred with the recognition of mutations by MutS, we tested the DNA target in human serum without MutS as well as DNA without mutations (i.e., a perfectly matched target). As expected, few spectral changes were observed in either experiment (Supplementary Fig. 5). After treating the microfluidic chambers at 95 °C, we detected the mutant target in serum solution containing MutS and observed identical red shifts, confirming that the fabricated single NP sensor preserved the specificity of MutS for DNA mutations.

### Sensitivity of sensing

We further investigated the sensitivity of the sNPS sensing method according to two parameters: the lowest concentration of MutS protein enabling an LSPR λ_max_ shift (Δλ_max_) to be effective within a certain detection time, and the LOD for analytes. After the MutS solution had arrived at the DNA-modified Au-bridged NPs in the microfluidic chamber, we allowed the reaction to continue for 1 min before obtaining RLS spectra for 10 s. An excess of DNA target was added to ensure complete hybridization with the probes. The effective concentration of MutS protein for the LSPR readout was 6.17 nM, corresponding to a 3.40 nm red shift in λ_max_ in the linear range of 10–25 nM MutS (Fig. 2e). We performed measurements using a blank sample and DNA targets at different concentrations to determine the LOD (Fig. 2f). The analytical range of 5–150 nM, where a plot of concentrations versus responses went linearly with an *R*^2^ of 0.9954, was observed, beyond which the linearity was inconsistent. The S/N was 9.86 while monitoring the 5 nM target. The LOD was calculated as 8.63 nM, which is comparable to that obtained with the label-free QCM method, and tens of fold lower than the value determined by label-free SPR bulk detection (Supplementary Note 1, Supplementary Fig. 14 and Supplementary Table 4)^14,16^. Most importantly, this high sensitivity was achieved at a flow rate of 1 μl min^−1^, and the total sample volume required for each detection was 30 μl with trace levels of sample. Excess and nonspecific materials were washed out of the microfluidic chamber and did not interfere with particle sensing. In contrast, fluorescence sensing methods require large amounts of reagent and many processing steps (e.g., MutS requires fluorophore modification at a pre-concentration >3 μM in buffers to achieve a labeling efficiency of <55%)^6,33^. A gel mobility shift assay requires loading of only a small volume, but samples must be highly concentrated for visualization^10,34^. Notably, the MutS footprint is 24 base pairs (bp), whereas interactions between protein and DNA are distributed over a large surface area (1250 Å^2^ or approximately 50 bp)^4^. The 51 bp ssDNA probe used in this study minimized signal loss due to nonspecific target binding and ensured clear differentiation between signal changes induced by different point mutations.

### Identification of single point mutations

**Fig. 3:**
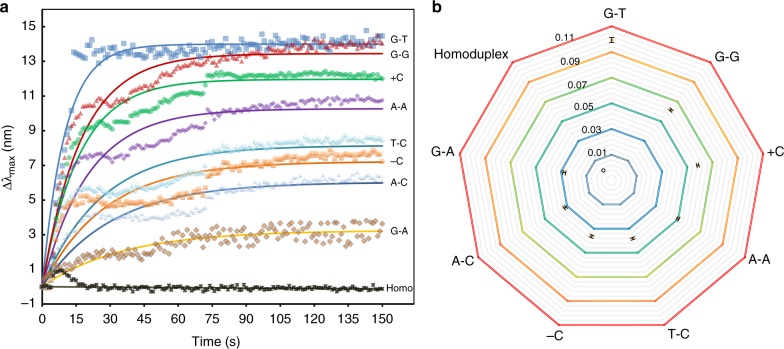
Identifiable detection of the eight single point mutations. **a** Real-time monitoring of MutS binding to each mutant DNA and a homoduplex. **b** Replotting of rate constants of interactions between MutS and each point mutation. Error bars represent mean ± S.E.M. All experiments were performed in triplicate. Source data are provided as a Source Data file

Point mutations are the most difficult to detect of all genetic alterations due to their subtle nature. We speculated that sequence-nonspecific binding of MutS to point mutations alter LSPR signals; to test this hypothesis, we examined the relative activity of MutS towards different nucleotide variants. We reviewed and selected eight most frequent polymorphisms from the spectrum of *BRCA1* mutations^35–39^, including six single-nucleotide substitutions (GT, GG, AC, TC, AA, and GA), an insertion (+C), and a deletion (−C). The DNA sequences, mutant names, genomic locations, functional consequences, and target populations are summarized in Supplementary Table 2. Upon injection into the sNPS chamber, MutS was allowed to bind to DNA-conjugated Au-bridged NPs for 150 s, and the changes in the optical response of a single NP were monitored every 1 s (Fig. 3a). Notably, MutS was loaded on homoduplex (perfectly matched) DNA for approximately 15 s according to real-time signal responses. This was consistent with a previous report that MutS forms a short-lived clamp and moves along homoduplex DNA by one-dimensional diffusional sliding, presumably in search of mismatched bases^4^. Mismatch identification resulted in a MutS binding time 10-fold longer than that of the homoduplex and induced serial Δλ_max_. Since this was caused by RI changes upon MutS binding to a DNA-conjugated NP, the time course clearly reflects the distinct activities of MutS in recognizing different point mutations, which presumably altered the contact between MutS and DNA, thus producing variable reaction constants in kinetic assays of MutS–DNA interactions.

We defined the relative activity of MutS to mutant DNA (*R*_act_) as the efficiency with which MutS binds to mutant DNA, expressed as *R*_act_ = *K* × *k*_reaction_, where *K* is an occupancy constant and *k*_reaction_ is the rate constant of the protein–DNA interaction^40^. This is a simple approximation of a stochastic binding event in which DNA on the Au-bridged NP is equally available for MutS; therefore, the same detection conditions allow the same *K* and *R*_act_ to be evaluated according to *k*_reaction_. The DNA probe length in this study (51 bp) implied 1:1 binding stoichiometry with MutS; thus, the time course of binding and disassociation can be described as a single exponential process. By fitting to the exponential equation, the *k*_reaction_ (10^−2^ s^−1^) values of MutS binding to different DNA targets were 9.95 ± 0.420, 6.15 ± 0.208, 5.80 ± 0.189, 4.92 ± 0.214, 3.82 ± 0.212, 3.60 ± 0.243, 3.25 ± 0.184, and 2.82 ± 0.197 for the point mutations GT, GG, +C, AA, TC, −C, AC, and GA, respectively. By replotting the *k*_reaction_ values as a function of each target DNA, the order of relative activity of MutS towards the mutations was determined as GT>GG>+C>AA>TC>−C>AC>GA (Fig. 3b), which is consistent with previous gel mobility shift assay data^10,34^.

### Reliability of sensing

**Fig. 4:**
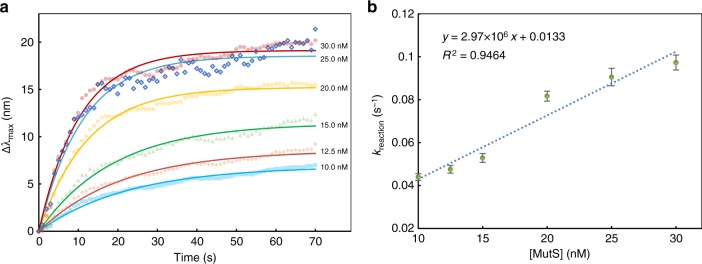
Reliability of sensing. **a** Real-time monitoring of MutS at various concentrations binding to GT-mutant DNA. **b** Dependence of rate constant on MutS concentration. Error bars represent mean ± S.E.M. All experiments were performed in triplicate. Source data are provided as a Source Data file

The crystal structure and interactions of MutS binding to a GT mismatch have been previously described in detail^20,32,41,42^. We therefore evaluated the reliability of the sNPS platform based on the *k*_reaction_ of MutS and GT-mutant DNA interaction. The kinetics of MutS binding to and dissociation from DNA can be described as *k*_reaction_ = *k*_binding_ [MutS] + *k*_dissociation_, where *k*_binding_ and *k*_dissociation_ are the binding and dissociation rate constants, respectively, and [MutS] represents the free molar concentration of MutS. [MutS] clearly affected reaction kinetics and corresponded to the amplitude of variation in Δλ_max_ (Fig. 4a). Higher [MutS] induced greater increases in λ_max_ prior to equilibrium, ultimately leading to longer shifts at the end of the interaction, which started at the maximum rate since no MutS had been consumed before the reaction slowed in a predictable manner to an equilibrium distribution of MutS. The rate constant *k*_reaction_ was quantitatively estimated by exponential fitting. Replotting *k*_reaction_ as a function of [MutS] yielded a linear equation (Fig. 4b) with *k*_dissociation_ as the *y* intercept and *k*_binding_ as the slope. The *k*_binding_ of 2.97 × 10^6^ M^−1^ s^−1^ was close to previously reported bulk kinetic measurements of 3–6 × 10^6^ M^−1^ s^−1 ^^6^. Kinetic studies of the ratio between *k*_dissociation_ and *k*_binding_ revealed the dissociation equilibrium constant of MutS to DNA—i.e., *K*_D_, a fundamental parameter of ligand affinity. The *K*_D_ of MutS was found to be 4.46 nM, which was in agreement with reported smFRET and bulk measurements of 2–20 nM^4,43^. This is the validation by kinetic studies of the equilibrium constant of MutS on a precise scale around a single NP, and supports the utility of the sNPS assay for biological applications.

### Atlas of MutS affinities to point mutations

**Fig. 5:**
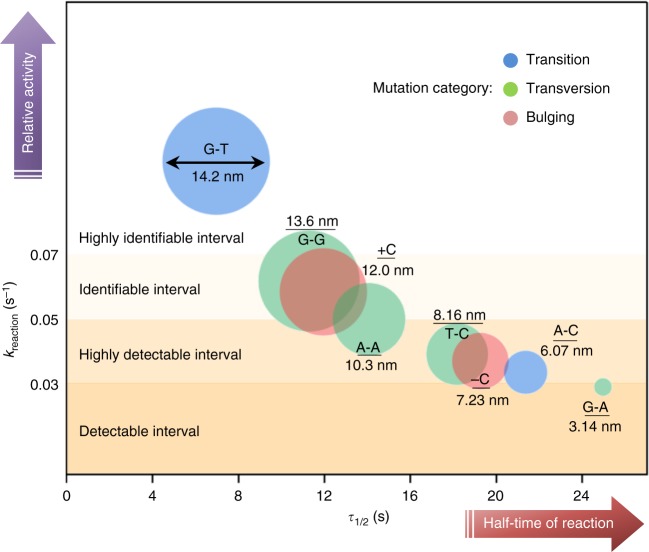
Atlas of MutS affinities to different point mutations. The atlas was established by single Au-bridged nanoparticle (NP) sensing a countable number of binding events between MutS and eight different mutations. Source data are provided as a Source Data file

We further established an atlas of protein binding affinities to DNA with four types of point mutation (Fig. 5): highly identifiable (*k*_reaction_ > 0.07), identifiable (*k*_reaction_ = 0.05–0.07), highly detectable (*k*_reaction_ = 0.03–0.05), and detectable (*k*_reaction_ < 0.03). The atlas shows comprehensive information obtained by low-input, high-fidelity sNPS of the relative activity and reaction half-time for each target, mutation type, and detection signal. Specifically, each circle represents a single point mutation, with the diameter and color reflecting the signal response for quantifying the LSPR peak shift and mutation category, respectively. For example, the target DNA with GT mutation generated a peak shift of 14.2 nm, the value of which is affected by and is in proportion to the concentration of targets (100 nM). The mutation category is biologically divided into three types, colored in blue, green, and red, of which the blue one indicates a transition mutation (replacement of a pyrimidine with a pyrimidine or vice versa), the green one indicates a transversion mutation (replacement of a pyrimidine with a purine or vice versa), and the red one indicates a bulging mutation (a base insertion or deletion). The *y* and *x* coordinates of the center of each circle represent the relative activity and half-time of the reaction, respectively. The relative activity predicted by *k*_reaction_ is dependent on the concentration of MutS used in the detections and the *K*_D_ of MutS to the target DNA. Therefore, single point mutations can be identified using MutS of the same quality and quantity by this sNPS method. The half-time of the reaction gives knowledge of the time it takes for MutS binding to the individual NPs to reach half of the maximum LSPR shift. Such knowledge can help to explain why, for instance, purine–purine mutations show better repair rates than pyrimidine–pyrimidine mutations in cells^44^, as evidenced by our observation that MutS bound more strongly to the purine–purine mutation (e.g., identifiable GG and AA) than to the pyrimidine–pyrimidine mutation (e.g., highly detectable TC). These results also indicate that repair of −C, AC, and GA mutations will be less effective since MutS has lower relative activity towards these than towards TC.

**Fig. 6:**
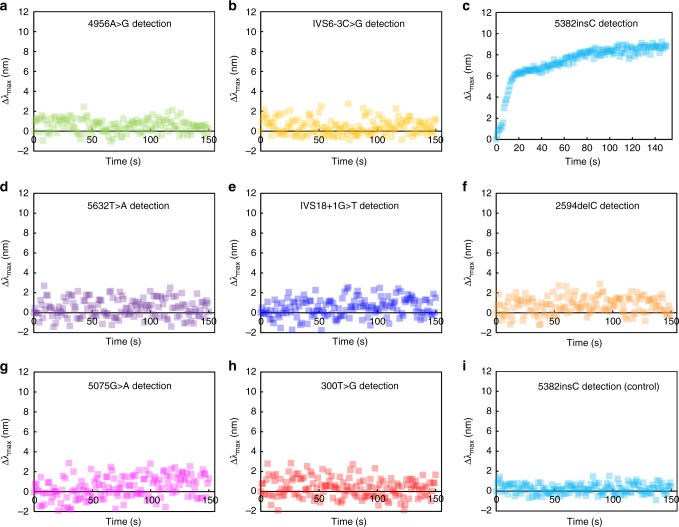
Detections of *BRCA1* point mutation with the eight single nanoparticle sensing (sNPS) chips developed in this study. **a**–**h** The sample from human breast cancer cell line, HCC1937, was detected as the analyte. None of the chips yielded an effective *k*_reaction_ except the 5382insC, indicating that the analyte contains a single cytosine duplication. **i** The sample from MCF7 was detected as the control. Source data are provided as a Source Data file

**Fig. 7:**
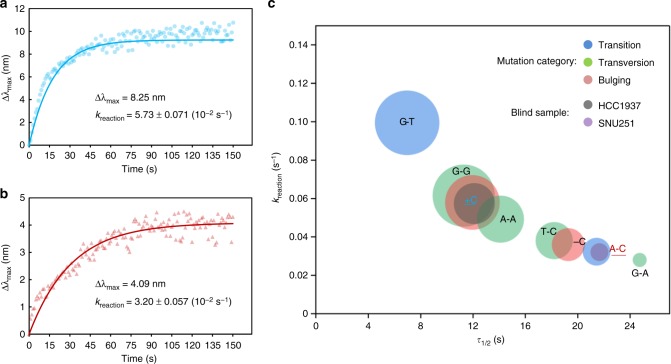
Analysis of the presence and type of potential mutations in *BRCA1* from human cancer cells. **a** Detection of the sample from breast cancer cells HCC1937. **b** Detection of the sample from ovarian cancer cells SNU251. **c** The *k*_reaction_ values in (**a**, **b**) were input into the atlas, predicting mutations of +C and A–C, respectively. Source data are provided as a Source Data file

As a proof-of-principle demonstration of clinical applications of the atlas, we prepared biological DNA samples from the human breast cancer cell lines, HCC1937 and MCF7, as an analyte and a control^45^, respectively, and detected the presence and type of a potential point mutation among the eight mutations shown in the atlas (Supplementary Figs. 6–8). Conspicuously, the glass chip designed for the detection of 5382insC (Supplementary Table 2) exhibited a series of peak shifts, while the other chips did not show significant signal variations (Fig. 6). Kinetic studies on the peak shifts in response to the detection time yielded a *k*_reaction_ of 5.73 ± 0.071 (10^−2^ s^−1^), which was in close proximity to the position of +C in the atlas (Fig. 7a, c), demonstrating that *BRCA1* of HCC1937 contains a single cytosine duplication. This result was confirmed by DNA sequencing (Supplementary Fig. 9). Additionally, the circle diameter of the target outputted the information of its concentration; for example, an 8.25 nm diameter indicated a 68.8 nM target according to the circle diameter (12 nm) of the +C in the atlas, which was obtained with a standard concentration of 100 nM.

Finally, we applied this sNPS system to detect potential point mutations in a user-assigned genomic region. A potential *BRCA1* point mutation located at 43047665 on region 2 band 1 of the long arm of chromosome 17 was assigned to test an ovarian cancer cell line, SNU251. We fabricated the chip with the same Au-bridged NP but with a new 64 bp probe. Interestingly, continuous shifts of the spectral peaks were observed, validating the effectiveness of the sNPS with the new probe to monitor a specific interval in the gene (Fig. 7b and Supplementary Fig. 10). Further, input of the detection results into the atlas indicated that the type of mutation was highly similar to AC point mutation (Fig. 7c). It is noteworthy that the *k*_reaction_ (10^−2^ s^−1^) yielded the similar values (3.20 in the detection versus 3.24 in the atlas), although the flanking sequences of the newly designed probe and the previously used AC probe were different. The small diameter of the purple circle indicated a low concentration of the target. The prediction of the AC mutation was confirmed to be accurate by DNA sequencing (Supplementary Fig. 11).

## Discussion

Our sNPS system enables low-input profiling of single point mutations in DNA by taking advantage of predesigned Au-bridged NPs. Plasmonics combined with scattering spectroscopy has contributed to advances in single-particle investigations by allowing light to be concentrated in plasmonic hotspots, while different nanostructures with bridges and gaps have been used in Raman and Rayleigh spectroscopy. Despite significant advances, these technologies have two critical challenges: (1) artificial signal reporters and high-energy laser concentration on NPs for Raman sensing hinder original probe–target interactions; and (2) predesigned plasmonic nanostructures are difficult to synthesize in solution. We used white light for label-free RLS from single particles, thereby preserving the physiological and biochemical conditions of the molecules. NPs with nanoscale bridges and gaps were obtained by direction-specific synthesis; this synthesis strategy controls the crystallization of Au atoms along the DNA framework from the nanoseed surface. In contrast to DNA or DNA origami-mediated metallization of polyploid structures, the products are colloidal crystalline particles comprising either nanobridges or nanogaps, which are adjustable by altering the ionic environment of the DNA interface. The nanobridge effectively increased refractometric plasmonic sensitivity for RLS, making the Au-bridged NP more sensitive than an AuNR of the same size. This demonstrates the DNA-directed synthesis-by-design of colloidal plasmonic nanostructures; the synthesized NPs can potentially be used for sensing and imaging biological interactions at high speed, resolution, and fidelity.

The implementation of the sNPS platform relies on microfluidic systems. On the scale of a few tens of nanometers, detecting a few molecules dispersed in a solution requires encounters with single NPs and their hotspots. Under ideal conditions, in which every biomolecule flowing through the detection channel must pass within the plasmonic field of the positioned single particle, the incubation time can be shortened from the current 15 min to real time, and the LOD can be further improved. Such nanofluidic devices can be manufactured by advanced nanofabrication techniques^46^. To count the number of biomolecules (*N*) in the sNPS, we investigated the effective space of plasmonic sensitivity to refractive index changes surrounding an individual nanoparticle, termed as plasmonic field of interest (FOI). The volume of the FOI (*V*) was calculated to be 0.953 μm^3^ (Supplementary Fig. 12). Here, the *N* can be obtained from the molar concentration of biomolecules (*C*) by *N* = *C* × *V* × *N*_A_, where *N*_A_ is the Avogadro’s constant. The lowest concentration of 6.17 nM protein in the Δλ_max_ confidence assays indicated that at least 3.5 MutS proteins in average must concurrently bind to the mutant DNA for a 3.40 nm red shift in λ_max_. The LOD value of 8.63 nM indicated that the detection was reliable after recognition of five strands of mutant DNA. The average number of probes (N^*^) loaded on an Au-bridged NP was estimated to be 207 by modeling of the nanoparticle and DNA footprint^47^ (Supplementary Fig. 13). The proportion of the probes completely capturing target DNA was hypothesized as 59.4%, corresponding to 215 nM of the saturation concentration for target detections. In consistence, the sensor signal was saturated at 200 nM (Fig. 2f). The 200 nM target binding is equal to 115 MutS molecules on the Au-bridged NP−DNA surface. In principle, loading all 115 molecules requires a minimum package volume of 7.12 × 10^−5^ μm^3^, which fits the available space of 9.91 × 10^−5 ^μm^3^ when the straight bar-like dsDNA is uniformly anchored on the NP as illustrated in Supplementary Fig. 12a. Thus, quantitative analysis confirmed that sNPS is useful for detecting small numbers of molecular binding events.

We predicted that the type of DNA point mutation was related to adaptations in base geometry and dynamics (Supplementary Note 2 and Supplementary Fig. 15). Mutations alter DNA geometry, with compensatory changes depending on the nature of the mismatched base and flexibility of the environment. These perturbations induce significant alterations in the ionic milieu of the DNA, which in turn influence DNA recognition by external factors such as the positively charged clamp domain of MutS. Additionally, the dynamic nature of mismatched bases affects the availability of DNA to proteins arising from the equilibrium between several different states of the bases. However, the correlation between the flanking sequence and the tautomeric or ionization state of DNA also affects its binding with MutS and potentially alter the kinetic parameters (e.g., Δλ_max_, LOD)^26^. In our analysis of the eight mutations, surrounding sequences were originally predetermined according to *BRCA1* and distinct from one another. To eliminate their influence, we used DNA probes that matched the MutS footprint. The design of probes should follow these principles: (1) the probe must guarantee one and only one MutS binding to potentially mutated targets by controlling its length to be shorter than 100 bp; (2) the probe must guarantee MutS binding near to the nanoparticle surface to sensitively generate peak shifts; and (3) the sequence of the probe needs to refer to the computed digestion maps of a target gene based on the available restriction enzymes. Given that MutS is in continuous rotational contact with duplex DNA followed by one-dimensional diffusion at the very high rate of approximately 700 bp s^−1^ to detect mutations^4^, the *k*_reaction_ of MutS binding to mutations of target DNA («700 bp) is expected to be specifically dominated by the following conformational dynamics: (1) base pair geometry, which induces changes in the tautomeric or ionization state of the bases; and (2) intermolecular interactions that undergo dynamic changes to modulate the orientations of nucleic and amino acids in DNA–MutS to achieve a mostly stable conformation. Therefore, the relative activity quantified by *k*_reaction_ in the atlas is more promising than an end-point result of peak shift for identifying a point mutation^21^. Long-term adaptions from a single mutation to surrounding bases and then to the active sites of a protein also suggest a mechanism by which MutS retains a memory of certain mutant conformations after binding^48^. Given that structural biological studies examining DNA–protein interactions provide insufficient information^49^ and that indirect reading mechanisms may play a role in defining MutS preferences^32^, it is difficult to unambiguously relate the distortions induced by mutations to the recognition ability of MutS. However, our data using synthetic DNA samples reveal the susceptibility of different mutations to recognition by MutS.

Gene mutations are associated with 10–30% of spontaneous cancers in a variety of tissues^50^. The sNPS technology described in this study provides a theoretical and experimental basis for analyzing interactions between mutant DNA and MutS for elucidating subtle variations in genes. Since the consistency of point mutation identifications between sensing and sequencing has been firmly established with cell lines, we believe that this sNPS system and the typical atlas will be useful for quick and reliable point mutation analysis in clinical applications. Our next step is to update the microfluidic units to fully integrate clinical sample processing^51^, nucleic acid extraction^52^, fragments of interest cutting^53^, and detection for rapid and sensitive sample-to-result genetic testing of clinical samples^54^. The introduction of another MMR protein, MutL, which can trap MutS at a DNA mismatch, will generate larger Δλ_max_ values for higher detection sensitivity; this could improve our understanding of the mechanisms by which MutS and MutL assemble on mismatched DNA for subsequent activation of MutH^6^. The fidelity to biomolecular interactions of this sensitive platform is also useful for investigating the bending kinetics of mutant DNA that control the activation of downstream signaling in the MMR system^33^.

## Methods

### Material information

Au nanoseed (AuNS; 5 nm) solution was obtained from British BioCell International (Crumlin, UK). Wash/storage buffers were from Ocean NanoTech (10 mM phosphate-buffered saline with 0.02% NaN_3_, 0.01% Tween 20, 0.1% bovine serum albumin, pH 7.4; catalog no.: WB-100, San Diego, CA, USA). Dithiothreitol (DTT) and restriction enzyme *Sty*I (#R648A) were from Promega (Madison, WI, USA). Microsep and Nanosep centrifuge tubes were from Pall Life Sciences (Ann Arbor, MI, USA). 2-{2-[2-(2-{2-[2-(1-Mercaptoundec-11-yloxy)-ethoxy]-ethoxy}-ethoxy)-ethoxy]-ethoxy}-ethylamine hydrochloride was from Cos Biotech (Daejeon, Korea). MutS protein derived from the thermophilic bacterium *Thermus aquaticus* was supplied by Nippon Gene Co. (Tokyo, Japan) and stored in 20 mM Tris-HCl buffer (pH 7.5) containing 100 mM NaCl, 0.1 mM EDTA, 1 mM DTT, and 50% glycerol at −20 °C. G-spin^TM^ Total DNA Extraction Kit (#17046) was supplied by iNtRON Biotechnology (Gyeonggi, Korea). Restriction enzymes *Mbo*I (#R0147) and *Alu*I (#R0137) were obtained from NEB (Hitchin, Hertfordshire, UK). Glycogen (#901393) was obtained from Roche (Indianapolis, IN, USA). Poly(ethylene glycol) methyl ether thiol (PEG, *M*_n_ = 800), hybridization buffer, hybridization wash pack, and all other chemical reagents were purchased from Sigma-Aldrich (St. Louis, MO, USA) and used without further purification. All glassware used in the experiments were cleaned in freshly prepared aqua regia solution and rinsed thoroughly with ultrapure water (18.2 mΩ cm^−1^) before use. All oligonucleotides used in this study were from Integrated DNA Technologies (Coralville, IA, USA). The sequences of the DNA targets and probes are shown in Supplementary Table 2. The assigned names and information of single-stranded DNA (ssDNA) are shown in Supplementary Table 3.

### NP modeling and numerical simulation

We performed modeling and optical simulations of nanostructures with spherical, rod, NP-gap, and bridged NP shapes using the commercial software COMSOL. NPs were composed of Au; particle sizes were set as uniform to facilitate comparisons. Optical simulations were performed in the local dielectric environment where water–glycerol mixtures of varying weight ratios were prepared so that the RI of the surrounding medium ranged from 1.333 to 1.443 (Supplementary Fig. 1).

### Conjugation of AuNSs with ssDNA

All 5’ thiol-modified oligonucleotides were incubated with DTT solution in a 1:100 ratio for 15 min and purified three times with ethyl acetate before use. The disulfide bond of the 5’-thiol was cleaved into an active sulfhydryl form and immediately conjugated with the Au surface. Before conjugation with DNA in solution, AuNSs were coated with bis(p-sulfonatophenyl)phenylphosphine dihydrate dipotassium (BSPP; 100 ml AuNS solution mixed with 100 mg BSPP for 10 h) to improve the tolerance of AuNSs to the highly ionic environment. The AuNS solution was then mixed with NaCl, which resulted in a color change from dark red to light violet. The solution was centrifuged for 30 min at 500 × *g* and the precipitate was resuspended in 1 ml of 0.5 mM BSPP. The solution again changed color from dark red to light violet upon addition of 0.5 ml methanol; the AuNSs were collected by centrifugation (30 min, 500 × *g*) and dissolved in 1 ml of 0.5× Tris–boric acid–EDTA (TBE) buffer. The concentration of AuNSs was increased to several μM, as measured with an ultraviolet (UV)–visible light–near-infrared spectrophotometer (UV-3600; Shimadzu, Kyoto, Japan); 1 OD of 5 nm AuNS is equal to 5.00 × 10^13^ particles per microliter according to the manufacturer’s instructions. The AuNSs were incubated overnight at room temperature with ssDNA-1 in a stoichiometric ratio of 1:1 in 0.5× TBE buffer containing 50 mM NaCl. The following day, 60% glycerol was added to the solution to obtain a final mixture of 10% glycerol to prevent AuNS-ssDNA from spreading in the buffers during gel electrophoresis. AuNSs with different numbers of bound ssDNA separated into different bands on a 3% agarose gel in 0.5× TBE buffer at 10 V cm^−1^ for 1 h (Supplementary Fig. 2a). The band corresponding to AuNSs conjugated with one strand of ssDNA (AuNS−1ssDNA-1) was incubated in 0.5× TBE buffer for further use. The procedures were carried out to conjugate AuNSs and ssDNA-2 to obtain AuNS−1ssDNA-2.

### Synthesis-by-design of NPs

Gold precursor (HAuCl_4_, 0.03%) and reductant (NH_2_OH·HCl, 1 mM) were separately dissolved in water and the pH of each solution was adjusted to 5 or 4 (±0.1) by gradually adding NaOH under a nitrogen environment. The seed for DNA-directed synthesis was produced by hybridization of AuNS−1ssDNA-1 with AuNS−1ssDNA-2 in the form of AuNS-dsDNA-AuNS. To increase hybridization efficiency, equal volumes of the two conjugates in 0.5× TBE were mixed and NaCl was added to increase ionic strength by 100 mM. The mixture was shaken overnight at 37 °C and the AuNS-dsDNA-AuNS was separated by gel electrophoresis with the same procedure of AuNS−1ssDNA-1 separation (Supplementary Fig. 2b). The gel containing AuNS-dsDNA-AuNS was soaked in 50 ml wash/storage buffer with a final PEG/seed molar ratio of 100:1. The solution was purified and concentrated by centrifugal tubes (molecular weight cut-off 30 K, 3000 × *g*), and thus the seeds were protected by the neutral PEG layer to improve stability and reduce the nonspecific absorption of charged molecules onto the AuNS surface. The seeds were gently stirred with gold precursor for 10 min at a final concentration of 2 nM; 10 μl of the solution was mixed with 17.54 μl reductant, and a color change from colorless to light-red was observed within 1 min. After 15 min, the synthesized NPs were washed by repeated resuspension in water and centrifugation. TEM and HR-TEM images of the NPs were obtained (HD2300; Hitachi, Tokyo, Japan) in z-contrast and secondary electron modes at an accelerating voltage of 300 kV. Samples were prepared for TEM using a staining plate (Electron Microscopy Sciences, Hatfield, PA, USA) and 400-mesh copper grids with carbon film (Ted Pella, Redding, CA, USA). The lengths and diameters of the nanostructures in the plane of TEM were measured using the software ImageJ. TEM images with scale bars of 20 nm and 50 nm showed nanostructures large enough for precise measurements. Particle yield was calculated as the ratio of Au-bridged NPs to total particles. The undesired particles were easily distinguished as oversized or undersized bridged-nanoparticles and as nanospheres grown from AuNS-ssDNA that were denatured from AuNS-dsDNA-AuNS during the synthetic reaction^28^. Fast Fourier transform patterns of HR-TEM images were analyzed with Digital Micrograph software (Gatan, Pleasanton, CA, USA) to confirm the crystalline structure and growth orientation.

### sNPS platform settings

The overall configuration of the sNPS system is shown in Supplementary Fig. 4a. To construct the detection chamber, microscope glass slides (22 × 40 × 0.1 mm) (Warner Instruments, Hamden, CT, USA) were coated with First Contact cleaning polymer (Photonic Cleaning Technologies, Platteville, WI, USA), which was immediately peeled off after curing for 15 min. The slide was rinsed overnight with freshly prepared aqua regia solution; after rinsing with ultrapure water, the slide was immersed in 5% (v/v) 3-aminopropyltriethoxysilane in absolute ethanol for 15 min followed by sonication in ultrapure water for 5 min. This process was repeated three times. A 3 μl volume of diluted Au-bridged NP solution (OD ~0.05) was added as a drop onto the silanized slide, followed by incubation for 1 min at room temperature. The slide was then washed with ultrapure water and ethanol in a sterile fumehood to prevent contamination with airborne debris before air drying with nitrogen gas. The slide was placed in a closed-bath imaging microfluidic chamber (RC-30; Warner Instruments) that was assembled onto a stage controller (Marzhauser Sensotech, Wetzlar, Germany) and connected to a flow device for solution mixing and a flow rate control system (PHD 2000; Harvard Apparatus, Holliston, MA, USA) (Supplementary Fig. 4b). Images of the field of view of the inverted microscope (Eclipse TE2000-U; Nikon, Tokyo, Japan) equipped with a 100 W halogen source (Type 7724, Philips), a dark-field dry condenser (NA = 0.80–0.95; Nikon), and 100× objective (CFI Plan Fluor ELWD, NA = 0.6; Nikon) were acquired with a color camera (D50; Nikon), and only individual nanoparticles with inter-particle spacing ~5-fold greater than the diameter of shinning dots were analyzed to minimize the effects of inter-particle resonance coupling (Supplementary Fig. 4c). Images from the chamber were focused on a charge-coupled device (CCD) camera (PIXIS: 400B; Princeton Instruments, Trenton, NJ, USA) at −70 °C with a 100 ms frame integration time. A beam splitter at the output port of the microscope and long-pass filter were placed before the CCD. The platform allowed the determination of RLS properties of each NP in the chamber using an RLS spectrograph (Microspec 2300i; Roper Scientific, Lisses, France) in a darkroom at 18 °C. Spectra in a range of 300–900 nm were recorded with acquisition time of 1 s. The spectral data were fitted with the Lorentzian algorithm to eliminate noise, and an accurate λ_max_ was determined using Origin2018 software (OriginLab, Northampton, MA, USA). Application of this method to analyze 10 spectra acquired once per minute from the same nanoparticle yielded a fitting-limited peak measurement precision of 0.188 nm (Supplementary Fig. 4e–f). The fluctuation in peak positions is attributed to instrumental factors including spectrometer resolution, physical uniformity in chambers, transient variations in temperature, and flow rate of liquid, and analytical factors such as microscope focus control, spectral source correction, exposed pixel selection, and spatial averaging. Besides the fluctuation of 0.188 nm, the total experimental peak uncertainty among random detections of 168 nanoparticles is 0.487 nm, where the 0.299 nm difference resulted from nanoparticle factors of size, shape, and orientation.

### Detection of point mutations

After mounting the glass slide in the sNPS system, the chamber was rinsed by injecting 75% ethanol for 5 min followed by rinsing with wash/storage buffer for 20 min to remove contaminants and unbound Au-NPs. The positions of Au-bridged NPs were recorded after photographing the chamber. One NP was representative to one detection set and its optical properties were determined for each step of molecule binding. The chamber was filled with 100 nM probe DNA (e.g., Probe-GT) for 8 h at room temperature and rinsed with wash/storage buffer for 5 min before introducing target DNA (e.g., 4956A>G) at different concentrations in hybridization buffer. Unbound target was removed by rinsing with the hybridization wash pack before injecting MutS solution at the target concentrations. The binding of MutS with DNA proceeded in binding buffer (100 mM NaCl, 1 mM DTT, 0.1 mM EDTA, and 5 mM MgCl_2_ [pH 7.5]) at a flow rate of 1 μl min^−1^ at 18 °C. For typical detection, 100 nM target DNA was captured by Probe-GT in the chamber and reacted with 20 nM MutS protein for 15 min. Real-time imaging of single NPs with a CCD camera and RLS spectra were recorded and processed using WinSpec software (Roper Scientific). Control experiments under the same detection conditions were conducted to investigate MutS interactions with probes (without target binding) and DNA homoduplex (homoDNA; without a mutation). For the investigations on DNA interactions with nonspecific proteins (without MutS), human serum was introduced after the injection of target DNA with GT mutations. After spectral analysis, the chamber was rinsed with wash/storage buffer at 95 °C for 30 min to remove the proteins. The same serum solution containing 20 nM MutS was injected into the chamber after capturing the same target, and then the spectra were recorded again for further analysis (Supplementary Fig. 5).

### Preparation and detection of samples from cell lines

We prepared biological DNA samples from the human cancer cell lines HCC1937 (American Type Culture Collection (ATCC), CRL-2336^TM^)^55^, SUN251 (Korean Cell Line Bank, 00251)^56^, and MCF7 (ATCC, HTB-22^TM^). MCF7 is an authenticated cell line used as control for *BACR1* analysis. All cell lines had no mycoplasma contamination. The genomic DNA was extracted using G-spin^TM^ Total DNA Extraction Kit and treated with 200 ng ml^−1^ proteinase K and 10 ng ml^−1^ RNase A at 55 °C for 30 min before purification and further restriction digestion. The digestion was performed with restriction enzymes *Mbo*I, *Alu*I, and *Sty*I to generate 50–60 bp nucleotides. In detail, digestion by *Mbo*I and *Alu*I yielded fragments of 100–500 bp. Since there are *Sty*I sites in *BRCA1*, the fragments were further digested by *Sty*I to the target sample of ~50 bp in length. The specific sites of the enzymes and the computed fragmentation maps can be found in Supplementary Figs. 6 and 7. To efficiently collect the DNA, glycogen was added during the ethanol precipitation following by centrifugation for 15 min at 10,400 × *g*. The concentration and purity of the DNA were assessed using a Nano-200 Micro-Spectrophotometer DC24V (#AS-11030-00; Allsheng Instruments, Hangzhou, China). The integrity of the DNA was evaluated by gel electrophoresis where 300 ng DNA samples were loaded onto 0.7% agarose gels at 2.5 V cm^−1^ at 4 °C, stained with 0.5% ethidium bromide, and detected by UV illumination with a Davinch-GelTM Gel Imaging System (Young Wha Scientific, Seoul, Korea) (Supplementary Fig. 8); 30 μl of the nucleotides was melted into single-stranded targets at 90 °C for 1 min and injected into the sNPS chamber filled with hybridization buffer at 70 °C for 5 min. Subsequently, the chamber was rinsed by the hybridization wash pack before introducing 20 nM MutS solution in binding buffer at a flow rate of 1 μl min^−1^ at 18 °C. For the detection of the sample from the SNU251 cell line, a new 64 bp probe was designed. Real-time RLS spectra were recorded and the peak positions were analyzed in the wavelength range of 500–650 nm with Lorentzian fitting.

### Demonstration of FOI of an individual NP

The plasmonic FOI of an individual nanoparticle is defined as the effective space of plasmonic sensitivity to refractive index changes, where Eq. 1 is applicable to calculate the molecular concentration in direct proportion to the amplitude of red shifts in λ_max_. The FOI was supposed to be cuboid (Supplementary Fig. 12a). The two-dimensional area of the FOI was directly delineated with 8 pixels in CCD images by the WinSpec software of the sensing system (Fig. 2d), the length and width of which were measured to be 3.36 μm based on the scale calibration (Supplementary Fig. 12b). We noted that the length and width of this two-dimensional area were set before the spectral monitoring and were maintained for all detections. As shown in the conformational scheme (Supplementary Fig. 12c), the height (*h*) of the FOI is the sum of the diameter (*D*_NP_) and the *t* of the nanostructure, where *t* is defined as the threshold thickness of the region that can induce peak red shifts. In detail, LSPR peak shifts exhibit an oscillatory behavior with a periodicity close to the *p* = λ_max_/2*n*, where *n* is the refractive index of the coating layer on the surface (Supplementary Fig. 12d)^57^. In the first half of the cycle, spectra exhibit red shifts with increasing thickness of less than *p*/2. Here, the threshold thickness of *p*/2 is *t*. Beyond the threshold thickness, the LSPR spectra begin to blue shift and then exhibit a periodic oscillation. Previous studies have demonstrated that the refractive index of the DNA layer (*n*_DNA_) is 2 and that the proteins and DNA behave similarly with respect to the refractive index change they induce^58–60^. Therefore, the *t* of Au-bridged NPs with an λ_max_ of 561 nm was calculated to be 70.1 nm. Finally, the volume of the FOI (*l* × *w* × *h* *=* *V*_FOI_) was established as 3.36 μm × 3.36 μm × 0.0844 μm = 0.953 μm^3^.

### Estimation of N^*^ per NP

The N^*^ was quantitatively predicted based on the modeling of the nanoparticle and DNA footprint (Supplementary Fig. 13). In detail, N^*^ was calculated by dividing the surface area of a particle by the area of effective footprint of a probe. The footprint is defined as the average area each probe occupies on the nanoparticle surface. Several assumptions were made for the calculation. The nanoparticle was modeled as two perfect spheres bridged by a cylinder; the footprints with the closest distance from each other were modeled as a circular area on the spheres and an ellipse on the cylinder; the contact-points of the two spheres on glass were not considered; and the probes were assumed to be evenly distributed on the particle surface.

The footprint area on the spheres (*S*_sphere_) is indexed to be 6 nm^2^ according to the diameter of the sphere^47^. The area of the two spheres (*A*_sphere_) was calculated by *A*_sphere_ = *A’*_sphere_ − *A*_contact_, where *A’*_sphere_ is the area of two separated spheres and *A*_contact_ is the contact area between the spheres and the cylinder; consequently, *A*_sphere_ = 2 × 4π(*D*_sphere_/2)^2^ – 2 × π(*D*_bridge_/2)^2^ = 1178 nm^2^, and thus the number of probes that can be packed on the spheres was N^*^_sphere_ = *A*_sphere_/*S*_sphere_ = 196.

The footprint area on the outer wall of the cylinder was calculated by the equation: N^*^_cylinder_ = n^*^_short-axis_ × n^*^_long-axis_, where n^*^_short-axis_ is the number of footprints around the circumference and n^*^_long-axis_ is the number down the axis of the cylinder. However, the length of the bridge (*L*_bridge_ = 2.39 nm) did not allow more than one row of probe loading along the axis of the cylinder because two rows on a non-curved surface would have a footprint spacing distance (4.72 nm as reported^47^) longer than 2.39 nm. Therefore, N^*^_cylinder_ = n^*^_short-axis_ × 1 = π*D*_bridge_/*l*_short-axis_, where π*D*_bridge_ is the circumference length and *l*_short-axis_ is the short axial length of the footprint given by *l*_short-axis_ = 2 × √[(3.3618 ln(*D*_bridge_/2) + 0.1616)/π]. The N^*^_cylinder_ was determined to be 11, and finally, N^*^ = N^*^_sphere_ + N^*^_cylinder_ = 207.

Due to the immobilization of the particle on a planar substrate, we hypothesized that only the surface above the line of edge of effective loading can effectively bind with DNA, which covers 59.4% of the total surface area of the particle (Supplementary Fig. 13). In a saturation condition, all the probes capture targets. Using the established equation of *N*_DNA_ = [DNA] *V*_FOI_ *N*_A_, the saturation concentration of the target DNA ([DNA]_saturation_) can be predicted by [DNA]_saturation_ = 59.4%N^*^/*V*_FOI_/*N*_A_ = 215 nM.

### Data analysis

Changes in RI corresponding to each molecular binding step on the NP surface are expressed as LSPR λ_max_ shifts (∆λ_max_):

1 $$\Delta \lambda _{{\mathrm{max}}} = m\left( {\Delta n} \right)\left[ {{\mathrm{1 - exp}}\left( {\frac{{{\mathrm{ - 2}}d}}{{L_d}}} \right)} \right],$$

where *m* is the refractive index sensitivity, Δ*n* is the change in refractive index induced by the adsorbate, *d* is the dielectric thickness and *L*_*d*_ is the electromagnetic field decay length (approximated as an exponential decay)^61^. The *m*, *L*_*d*_, and *d* are invariables of the sNPS system for the same nanoparticles and the same lengths of probes and proteins; and therefore, ∆λ_max_ is in direct proportion to Δ*n*, which is proportional to the concentration of the bound analytes^62^. Based on the measurements of ∆λ_max_, the changes in concentrations of the analytes were calculated.

The lowest concentration of MutS protein yielding a reliable Δλ_max_ was determined as the limit of quantification of the sNPS procedure as follows:

2 $${\mathrm{Limit}}\,{\mathrm{of}}\,{\mathrm{quantification = 10}}{\it{\sigma }}{/S},$$

where *σ* is the standard deviation of the signal and *S* is the slope of the calibration curve. The value of *σ* was estimated from the standard deviation of the *y* intercept of the regression line.

The LOD of the sNPS system for DNA target was determined as follows:

3 $${\mathrm{LOD = 3}}{\mathrm{.3}}{\it{\sigma }}{/S}.$$

The S/N was defined as the ratio of the mean (*μ*) to the standard deviation of Δλ_max_. An S/N of 5 is the threshold value to distinguish signals at 100% certainty^63^.

4 $${\mathrm{S/N = }}{\it{\mu }}{\mathrm{/}}{\it{\sigma }}.$$

In the protein-nucleic acid binding reaction, MutS binds DNA, forming the MSDNA complex:

5 $${\mathrm{MutS}} + {\mathrm{DNA}} \rightleftharpoons {\mathrm{MSDNA}}.$$

Conceptually, both the binding and dissociation reactions involve straight binding. At the level of a single DNA strand, MutS association and dissociation are stochastic processes. By simple approximation, all DNA strands on the Au-bridged NP are equally available for binding. The lengths of DNA strands used in this study indicate binding in a 1:1 stoichiometry with MutS; the time course of binding is described by a single exponential process. At the steady state, the rate of binding is equal to the rate of release:

6 $$k_{{\mathrm{binding}}}\left[ {{\mathrm{MutS}}} \right]{\mathrm{[DNA]}} = k_{{\mathrm{dissociation}}}{\mathrm{[MSDNA]}},$$

where [MutS] and [DNA] are the free molar concentrations of MutS and DNA, respectively; and *k*_binding_ and *k*_dissociation_ are the association and dissociation rate constants, respectively.

Before reaching the steady state, the rate of change of [MSDNA] is equal to the difference between its formation and dissociation rates:

7 $${\mathrm{d[MSDNA]}}/dt = k_{{\mathrm{binding}}}\left[ {{\mathrm{MutS}}} \right]\left[ {{\mathrm{DNA}}} \right] - k_{{\mathrm{dissociation}}}{\mathrm{[MSDNA]}}.$$

The binding starts at the maximum rate and then slows in a predictable manner as reactants are consumed. The extent of the reaction over time can be expressed as follows:

8 $$[{\mathrm{MSDNA}}]{\mathrm{ = [MSDNA}}_{{\mathrm{max}}}{\mathrm{] - [MSDNA}}_{{\mathrm{max}}}{\mathrm{]e}}^{ - (k_{{\mathrm{binding}}}[{\mathrm{MutS}}]{\mathrm{ + }}k_{{\mathrm{dissociation}}})^t}{\mathrm{ + [MSDNA}}_{t_0}{\mathrm{]}}.$$

The initial concentration of MSDNA ([MSDNA_*t*0_]) was zero and hence the above equation can be transformed into the following:

9 $$[{\mathrm{MSDNA}}] = [{\mathrm{MSDNA}}_{{\mathrm{max}}}]\left( {{\mathrm{1}} - {\mathrm{e}}^{ - k_{{\mathrm{reaction}}}t}} \right),$$

where $k_{{\mathrm{reaction}}} = k_{{\mathrm{binding}}}\left[ {{\mathrm{MutS}}} \right] + k_{{\mathrm{dissociation}}}$ is the observed reaction rate constant. The ratio of *k*_dissociation_ and *k*_binding_ yields the equilibrium constant (*K*_D_, in nM) of MutS protein, which was used to evaluate the strength of bimolecular interactions and is calculated with the following equation:

10 $$K_D = \frac{{k_{{\mathrm{dissociation}}}}}{{k_{{\mathrm{binding}}}}}.$$

Further transformation of the Eqs. (9) and (10) can get the equation:

11 $$k_{{\mathrm{reaction}}} = k_{{\mathrm{dissociation}}}\left( {\frac{{\left[ {{\mathrm{MutS}}} \right]}}{{K_D}} + 1} \right),$$

where *k*_dissociation_ is independent of concentration and indicates the probability that the complex will spontaneously fall apart in a unit of time^64^.

Based on time courses of the λ_max_ change, the time for bindings to reach half of the maximum ∆λ_max_ was evaluated by the half-time of the reaction (*τ*_1/2_):

12 $$\tau _{{\mathrm{1/2}}} = \frac{{{\mathrm{ln}}2}}{{k_{{reaction}}}}.$$

### Reporting summary

Further information on experimental design is available in the Nature Research Reporting Summary linked to this article.

## Supplementary information

Supplementary Information

Reporting Summary

Source Data file

## Data Availability

The data supporting the findings of this study are available within the article and its Supplementary Information. The source data of Figs. 1b, d, 2b, c, e, f and 3–7, and Supplementary Figs. 4e, 5 and 10 are provided as a Source Data file. All data are available from the corresponding author upon reasonable request.
