# Supplementary material for: Single gold-bridged nanoprobes for identification of single point DNA mutations
Source: Nat Commun. 2019 Feb 19;10:836. doi: 10.1038/s41467-019-08769-y (PMC6381086; doi:10.1038/s41467-019-08769-y)
Supplement: Supplementary file 1 — Supplementary Information [file 41467_2019_8769_MOESM1_ESM.pdf]

Single gold-bridged nanoprobes for identification of single point DNA mutations

Ma *et al.*

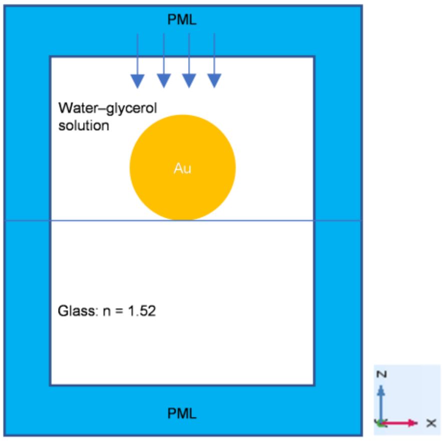

Supplementary Figure 1. Schematic illustration of RI sensitivity analysis of single NPs. LSPR wavelength shifts in response to changes in RI of the surrounding medium. The RI values were set to be 1.44290, 1.41299, 1.38413, 1.35749, and 1.33300, respectively, for the medium compositing 80%, 60%, 40%, 20%, and 0% glycerol in water.

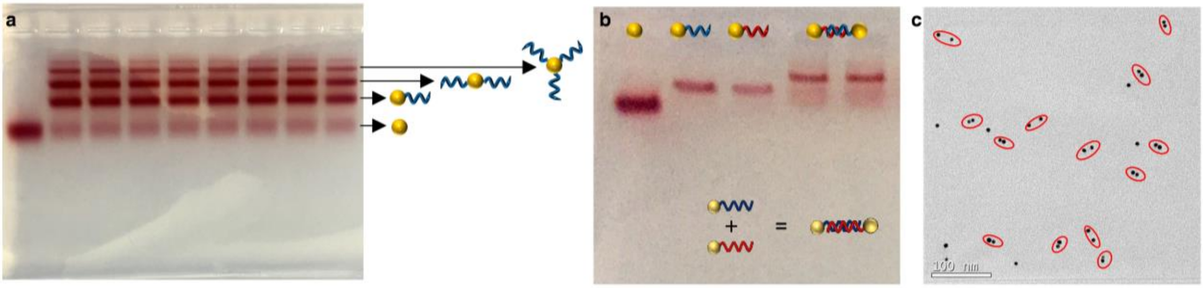

Supplementary Figure 2. Preparation and characterization of AuNS-dsDNA-AuNS. a, Image of a gel of different AuNS-ssDNA conjugates separated by electrophoresis. Each band represents a certain number of ssDNAs anchored to one AuNS. The leftmost band shows the bare nanoseed without binding of DNA. **b**, Image of a gel of AuNS-dsDNA-AuNS separated by electrophoresis after ssDNA hybridization. **c**, TEM image of AuNS-dsDNA-AuNS.

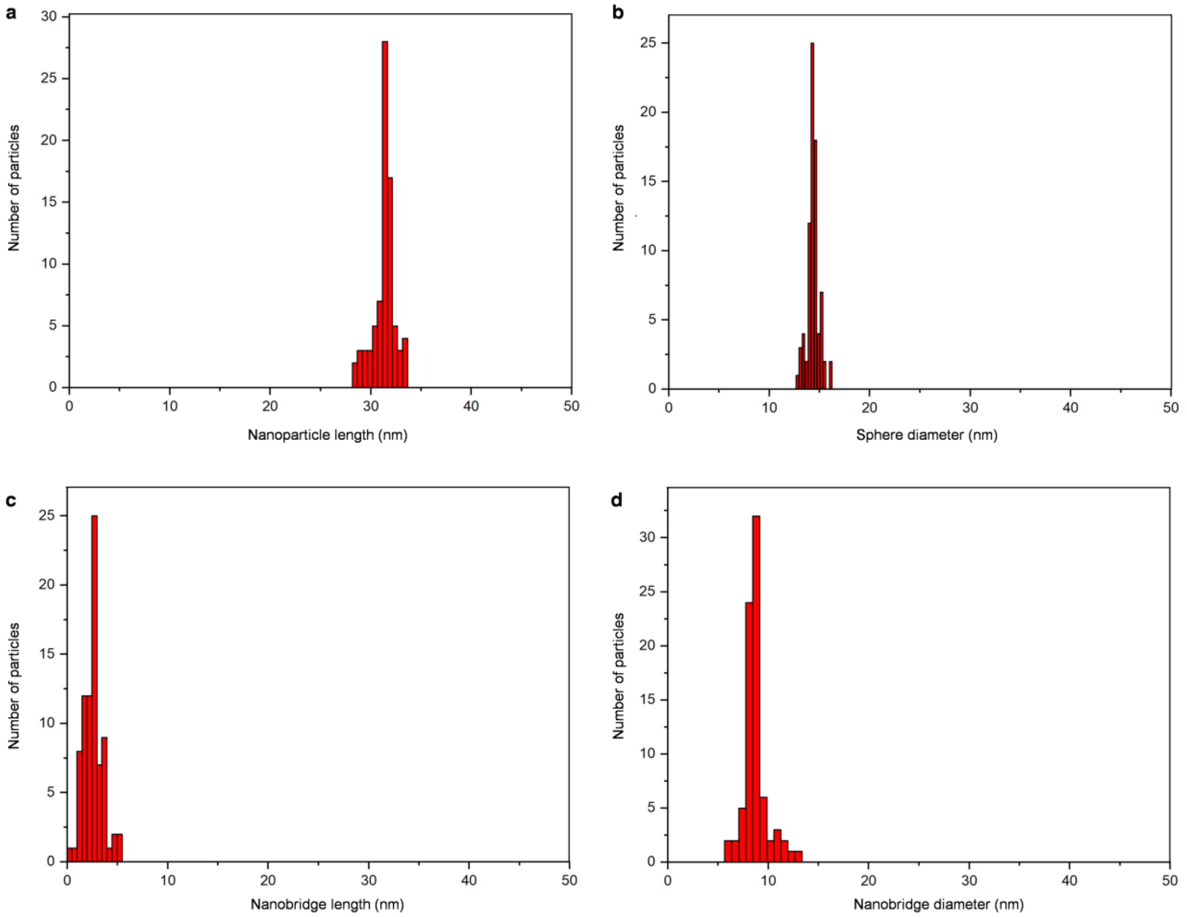

Supplementary Figure 3. Size distribution of Au-bridged NPs. Lengths and diameters of the nanostructures were measured using ImageJ. The statistical results demonstrated narrow-sized distributions.

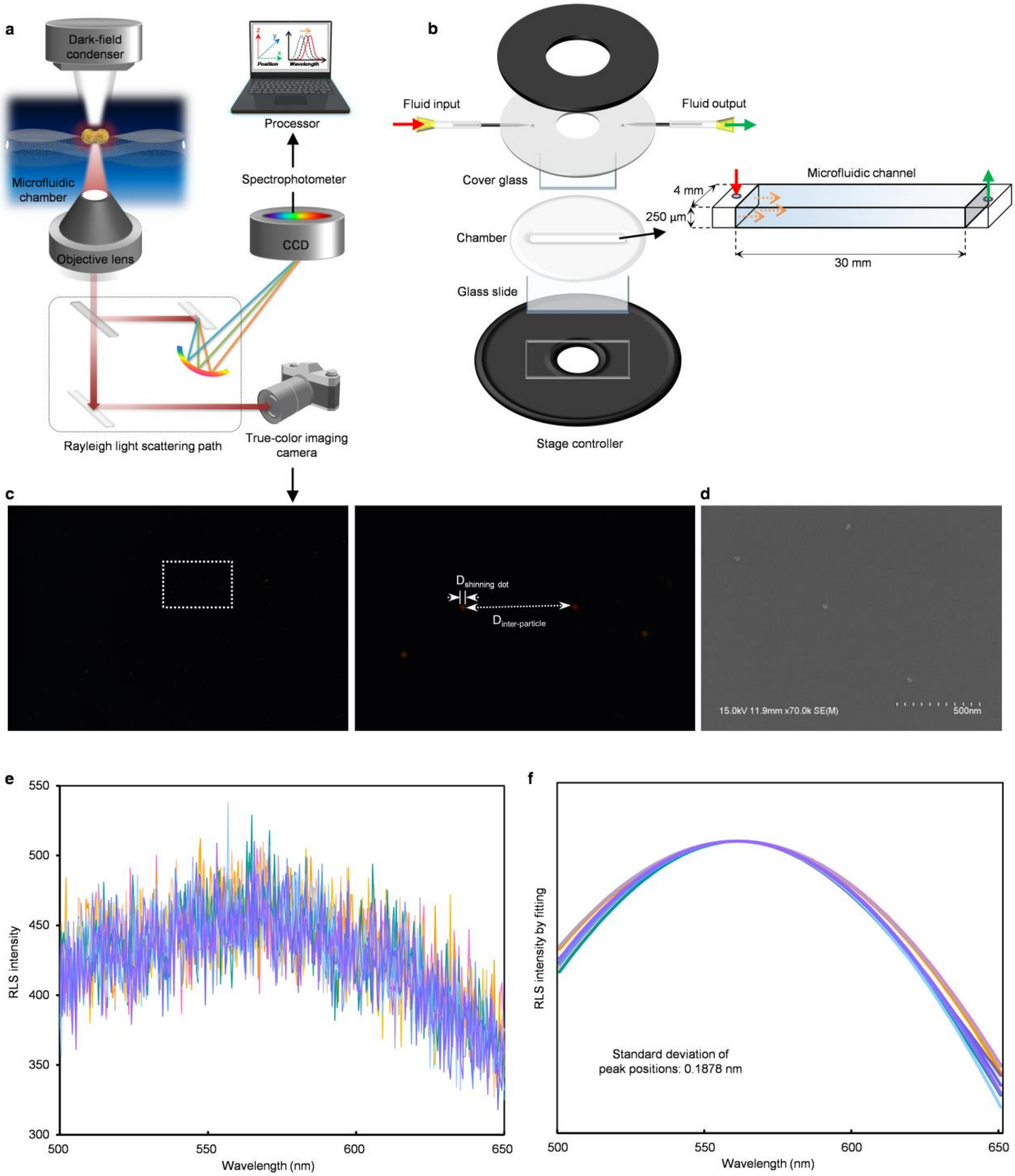

Supplementary Figure 4. sNPS system. **a**, Detailed configuration of the sNPS system based on RLS and LSPR of single Au-bridged NPs by white-light irradiation. **b**, Schematic diagram of the detection chamber. **c**, Image of the chamber acquired by the camera. Individual nanoparticles with inter-particle spacing ~5-fold greater than the diameter of shinning dots were position-marked and analyzed. **d**, SEM image of the chamber. **e**, Raw spectra of an Au-bridged NP acquired once per minute for 10 min. **f**, Lorentzian fitting of the 10 raw spectra demonstrated a peak measurement precision of 0.188 nm.

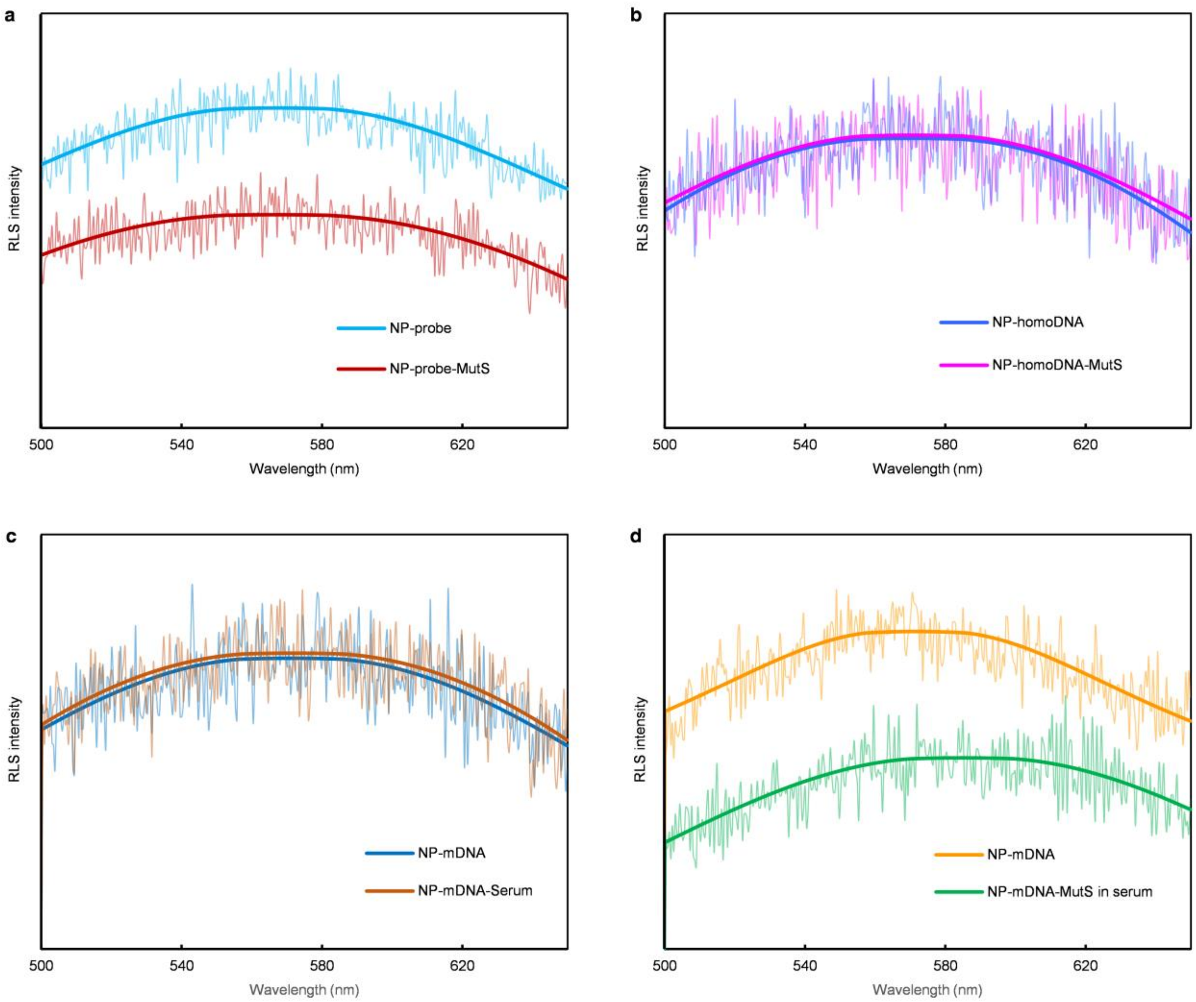

Supplementary Figure 5. Sensing specificity. **a**, Control experiment of MutS with probes. RLS spectra of a single NP showed no significant λ_{max} shift (0.356 nm blue-shift). **b**, Control experiment of MutS with homoDNA showing 0.343 nm red-shift in λ_{max}. **c**, Control experiment of mutant target DNA (mDNA) with nonspecific components in serum. RLS spectra showed no significant λ_{max} shift (0.700 nm red-shift). **d**, Introduction of MutS into the serum significantly generated a 14.7-nm red-shift under the same detection conditions as in **c**.

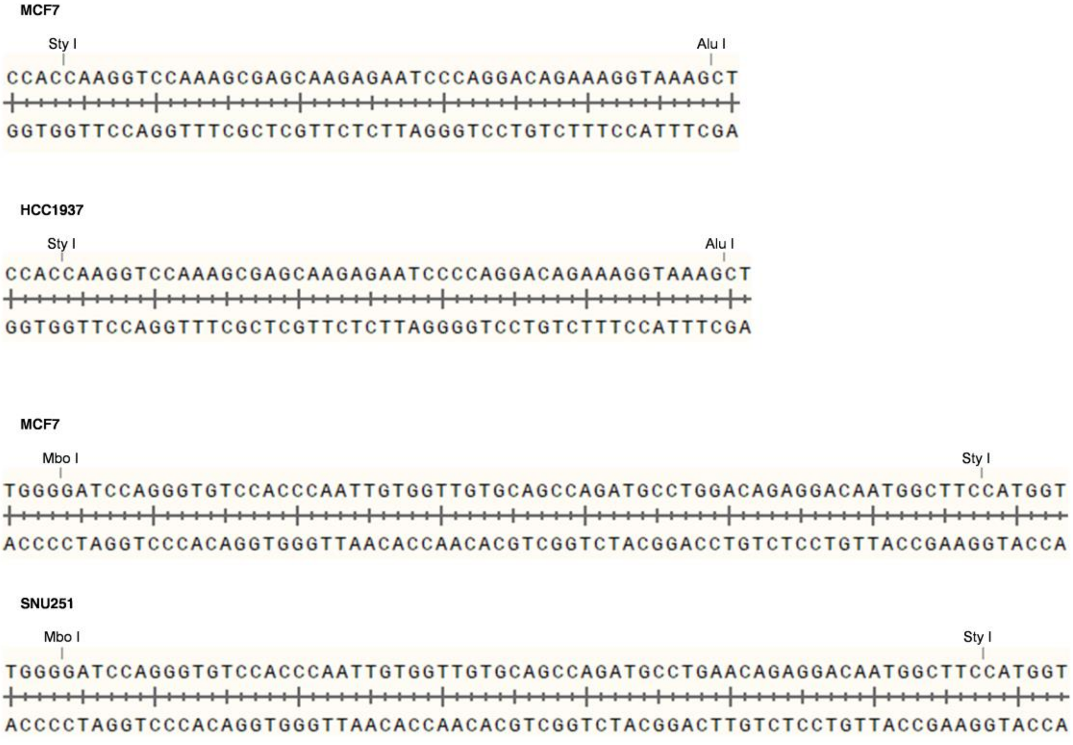

Supplementary Figure 6. Sequences showing the active sites of the restriction enzymes. *Mbo*I, 5’–GATC–3’; *Alu*I, 5'–AGCT–3’; *Sty*I, 5'–CCWWGG–3'.

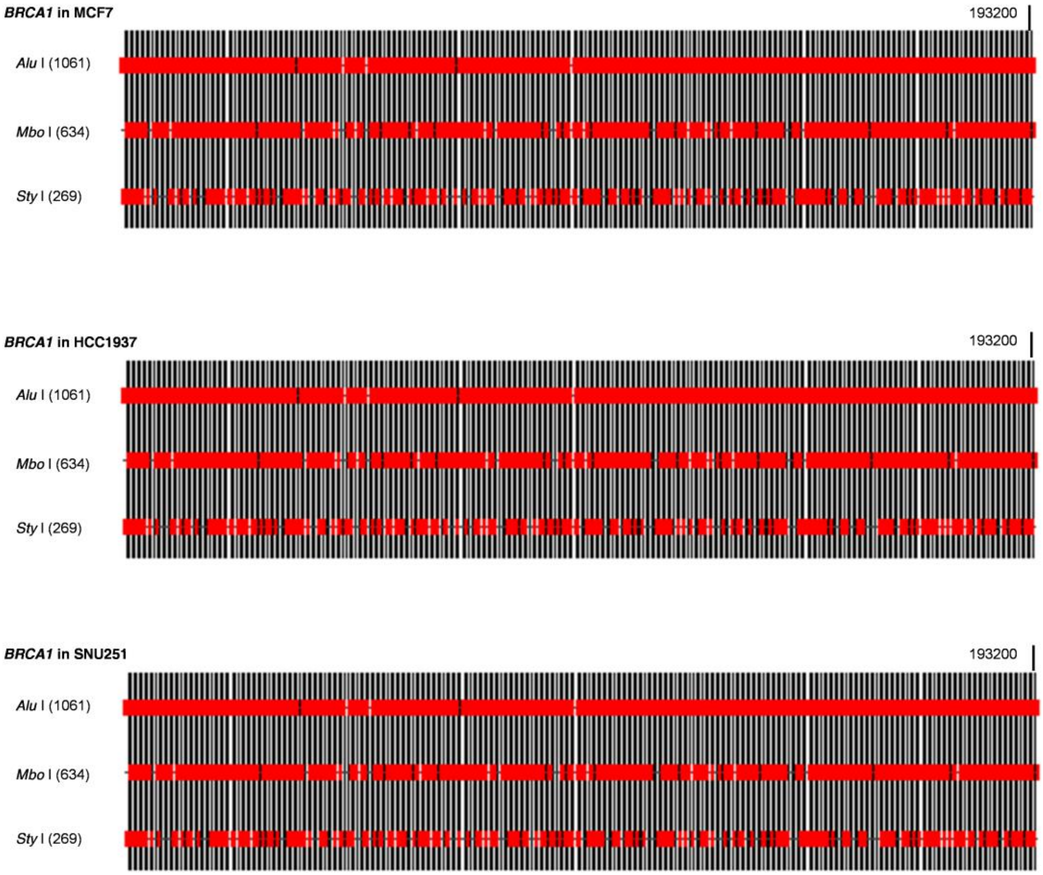

Supplementary Figure 7. Computed fragmentation maps of *BRCA1* of different cell lines after restriction digestion by enzymes *Mbo*I, *Alu*I and *Sty*I. The software GENETYX 4.0 (Genetyx Corporation, Tokyo, Japan) was used for the analysis.

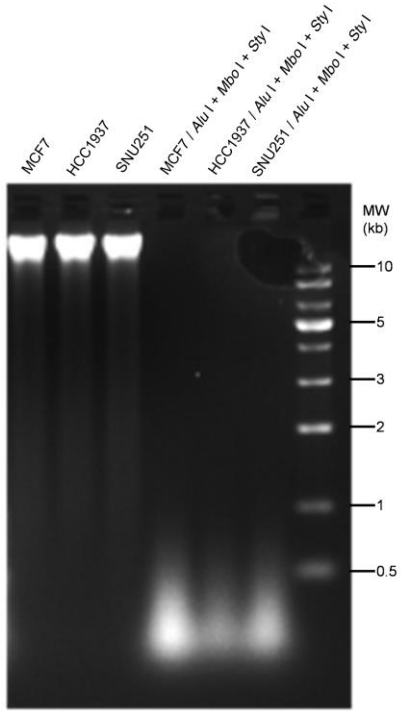

Supplementary Figure 8. Agarose gel electrophoresis of *BRCA1* DNA before and after synergetic digestion by three restriction enzymes (DNA ladder shown in the rightmost). The genomic DNA was extracted from breast cancer cell lines MCF7 and HCC1937, and an ovarian cancer cell line SNU251.

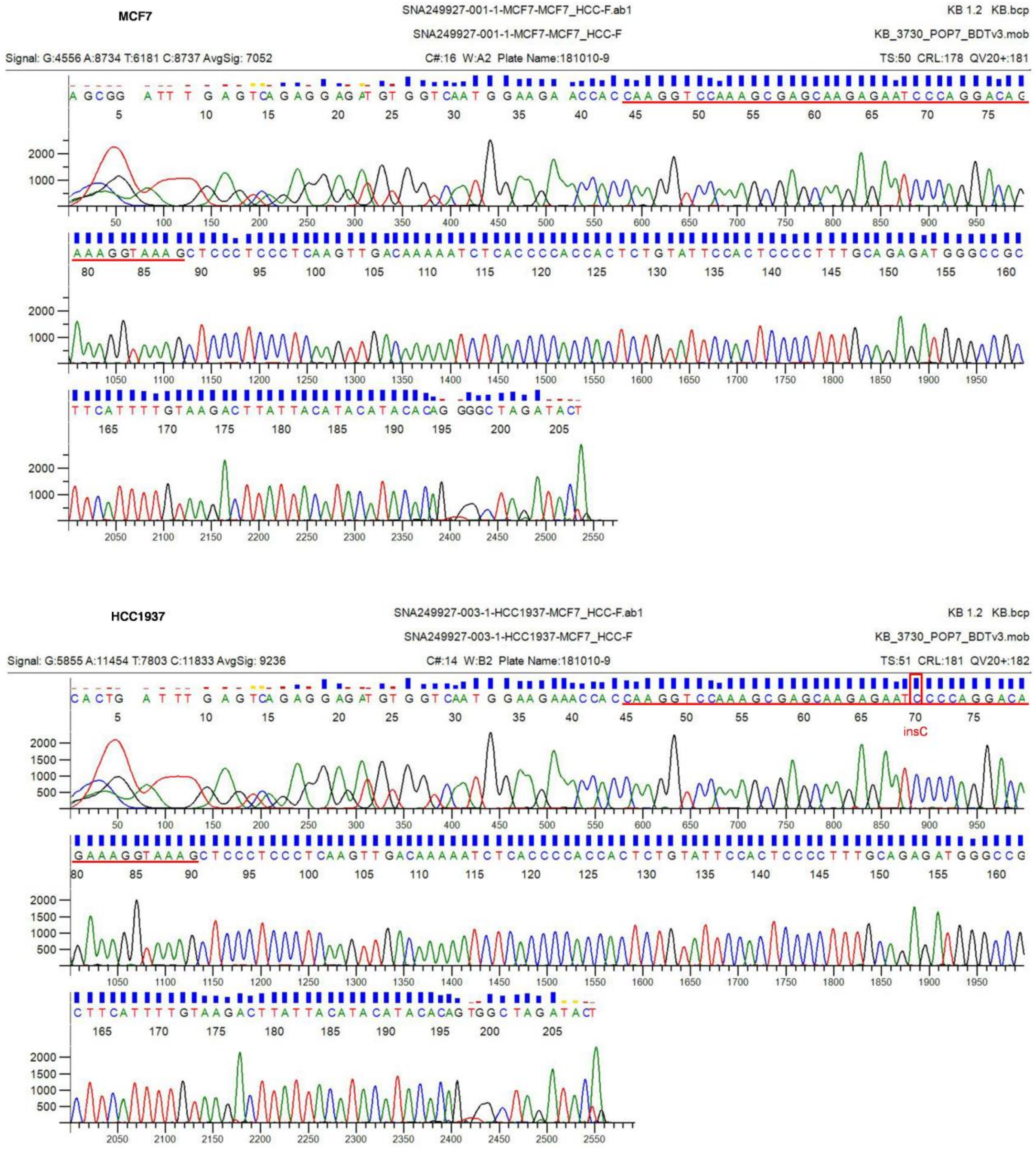

Supplementary Figure 9. DNA sequencing results. Compared with the *BRCA1* sequence of the cell line MCF7 (negative control without a point mutation), the sequence of HCC1937 exhibited a single cytosine insertion.

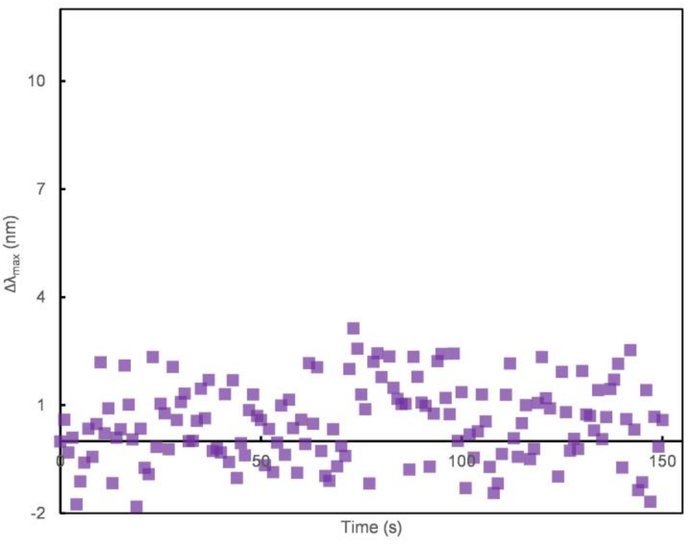

Supplementary Figure 10. Control experiments of detecting samples from MCF7 cells for the analysis of point mutation in the user-assigned genomic region of *BRCA1*.

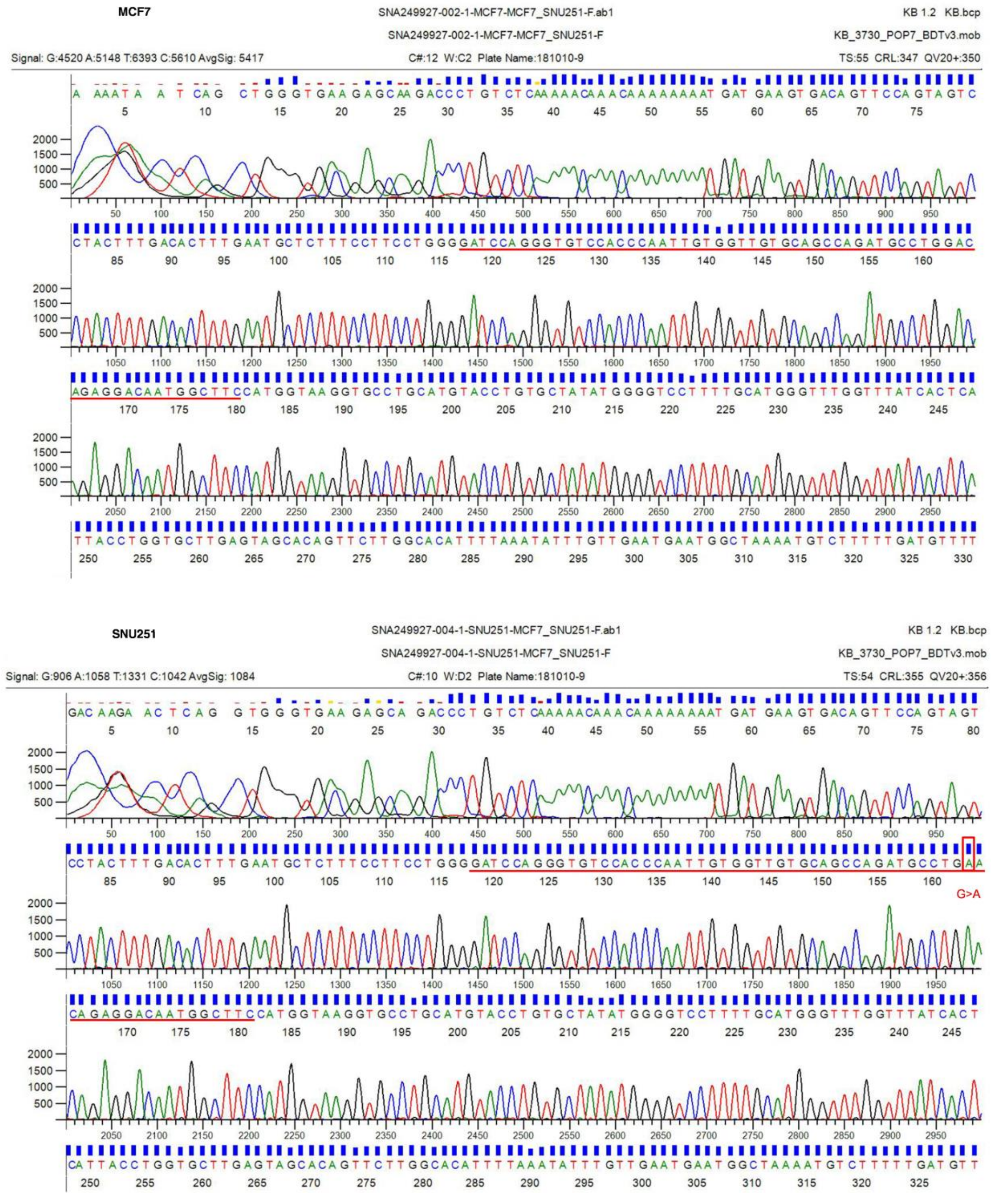

Supplementary Figure 11. DNA sequencing results. Compared with the *BRCA1* sequence of the cell line MCF7 (negative control without a point mutation), the sequence of SNU251 exhibited a single G>A substitution.

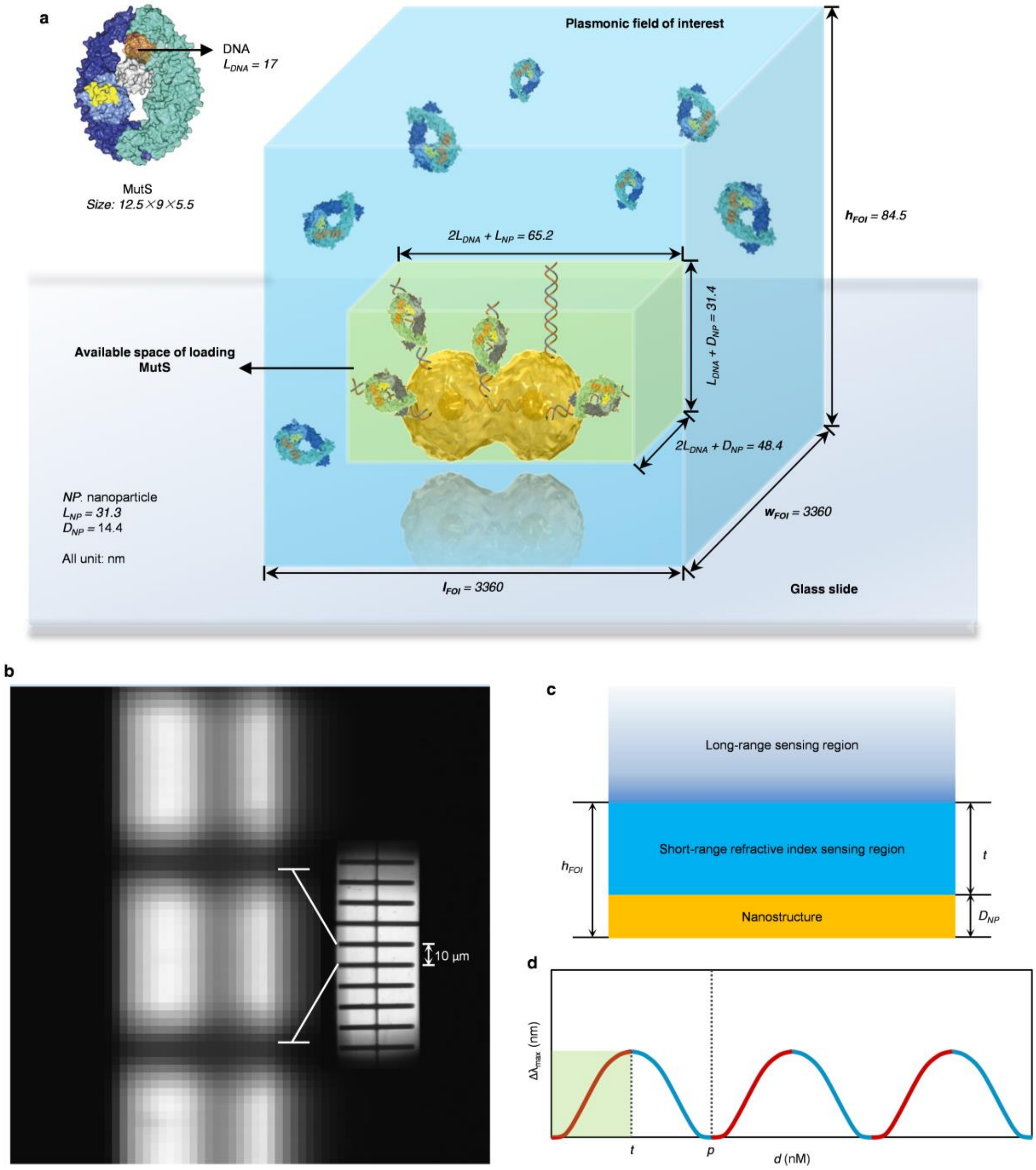

Supplementary Figure 12. Plasmonic field of interest (FOI) of an individual nanoparticle. a, Schematic diagram of the FOI and the available space for loading MutS of an Au-bridged NP. **b**, Scale calibration of the CCD images. The length of one pixel is equal to 0.42 μm. **c**, Illustration of height (*h*) of the FOI. **d**, Illustration of the periodicity of LSPR peak shifts. The region marked in green color is the short-range refractive index sensing region where the equation, $\Delta\lambda_{max} = m\ \Delta n\ [1 - \exp(-2d/L)]$, can be used to describe LSPR wavelength shift as a function of the medium concentration changes. Since this equation does not apply to long-range LSPR sensing, the schematic curve does not consider real profiles of the oscillation behavior in the long-range LSPR (e.g. inharmonic properties).

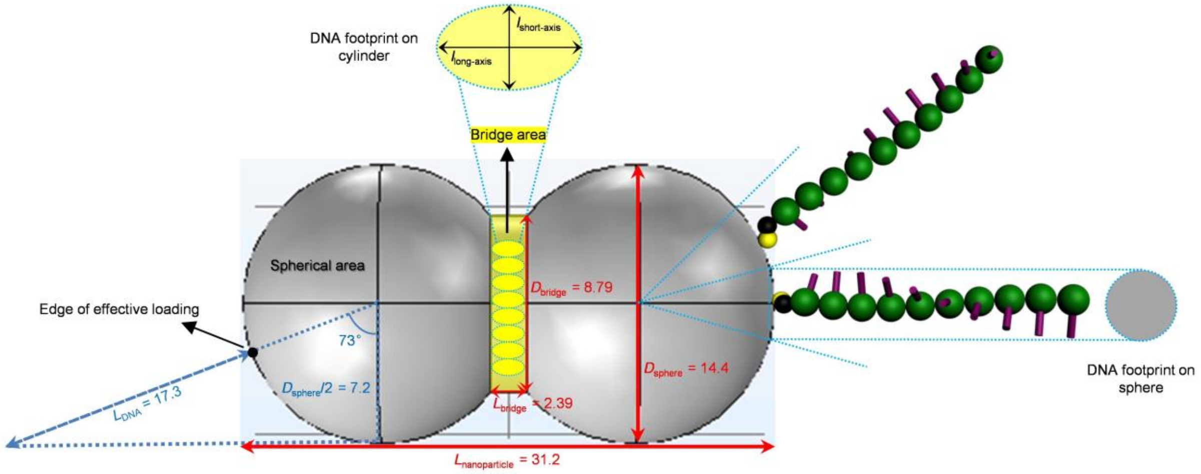

Supplementary Figure 13. Estimation of the average loading number of probes (N^*) per Au-bridged NP. The nanoparticle was modeled as two spheres (gray color) bridged by a cylinder (yellow color); the DNA footprints were assumed to be evenly distributed on the particle with the highest loading density and the closest distance from each other, and thus, were modeled as a circular area on the spheres and an ellipse on the cylinder. The ratio of the surface area above the line of "edge of effective loading" to the total area approximates to (180° - 73°)/180° = 59.4%.

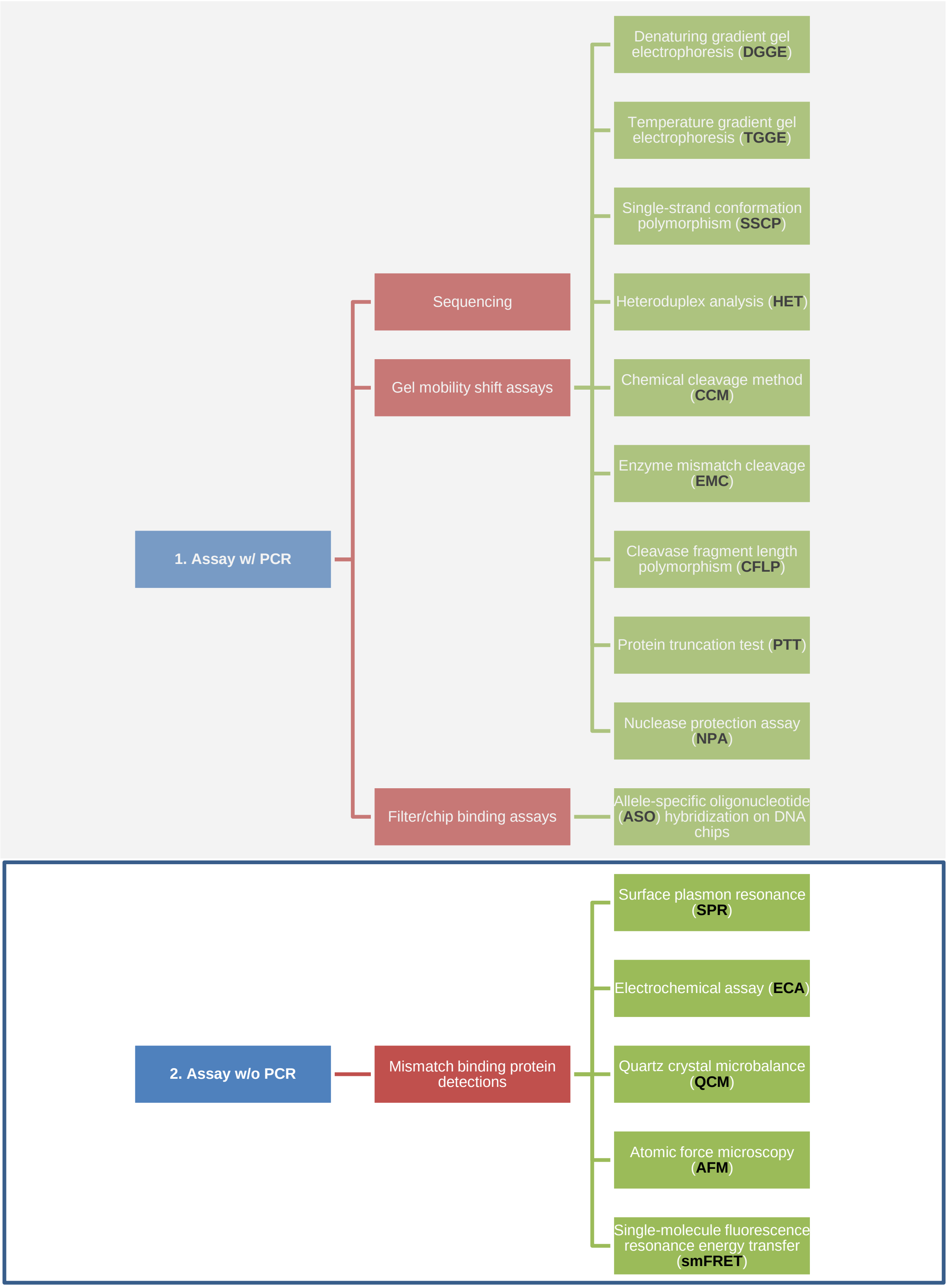

Supplementary Figure 14. Summary of currently used methods for detection of single point mutations.

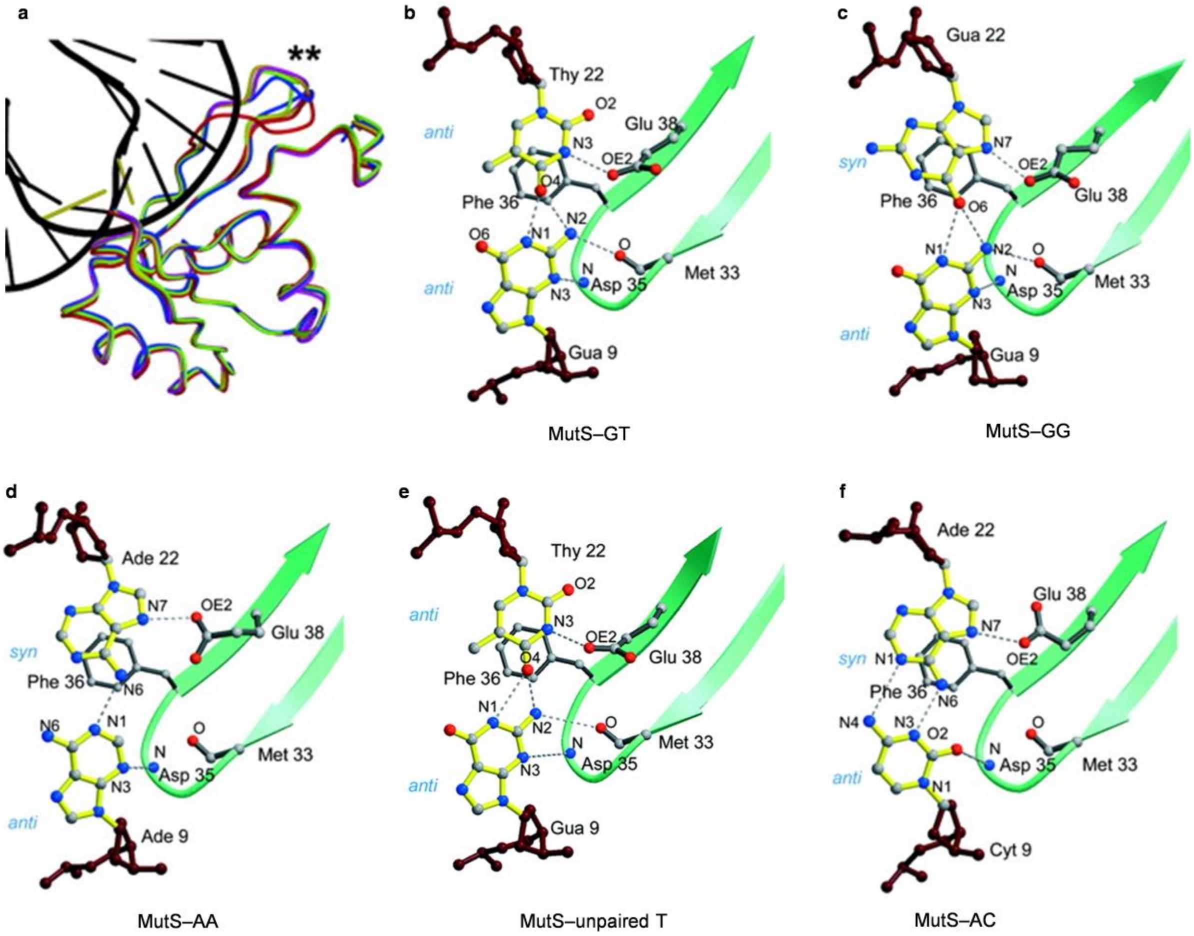

Supplementary Figure 15. Structural study of DNA binding by MutS[1]. a, Superposition of the mismatch binding domains of MutS. The variation in the loop between Ala 60 and Gly 63 is indicated by the two asterisks. **b**, GT mismatch bound to MutS[2]. **c**, GG mismatch bound to MutS[3]. **d**, AA mismatch bound to MutS[1]. **e,** Unpaired T bound to MutS[4]. **f**, AC mismatch bound to MutS[5].

Supplementary Table 1. Dimensional information of Au-bridged NPs

**Au-bridged nanoparticles**	**Length (nm)**		**Diameter (nm)**	
	Particles	Nanobridges	Spherical ends	Nanobridges
Mean	31.15	2.39	14.38	8.79
Standard deviation	1.00	0.87	0.58	0.96
Dimensional deviation[a]	3.20%	36.5%	4.03%	10.9%
p-value[b]	0.9912	0.9786	0.9522	0.9897

[a]Dimensional deviation is the ratio of the standard deviation to the average size.
[b]Nanoparticles were obtained from 8 batches of synthesis and 194 particles in the plane of TEM images were analyzed.

Supplementary Table 2. Information of important and common point mutations of *BRCA1* for detections

Mutation name[a]	Genomic location[b]	Allele ID	Nucleotide variant type	Base pairing	Functional consequence	Populations	DNA sequence[c]	Probe sequence[d]
4956A>G	GRCh38, 17:<br>43071077..43<br>071077	50266	Single<br>substitution<br>A>G	G*-T	Protein changes:<br>S1613G, S1634G,<br>S509G	Worldwide	ATTGAAAGTTGCAGAATC<br>TGCCCAGGGTCCAGCTGC<br>TGCTCATACTACTGA	TCAGTAGTATGAGCAGCA<br>GCTGGACTCTGGGCAGAT<br>TCTGCAACTTTCAAT
IVS6-3C>G	GRCh38, 17:<br>43104264..43<br>104264	46057	Single<br>substitution<br>C>G	G*-G	Anomalous<br>splicing leading to<br>premature<br>translation and<br>truncated protein	Worldwide	ACATAATGTTTTCCCTTGT<br>ATTTTAGAGATGCAAACA<br>GCTATAATTTTGCA	TGCAAAATTATAGCTGTTT<br>GCATCTGTAAAATACAAG<br>GGAAAACATTATGT
5075G>A	GRCh38, 17:<br>43070958..43<br>070958	50269	Single<br>substitution<br>G>A	A*-C	Protein changes:<br>M1652I, M1625I,<br>M548I, M1673I	Worldwide	AAAGGGTCAACAAAAGA<br>ATGTCCATAGTGGTGTCTG<br>GCCTGACCCCAGAAG	CTTCTGGGGTCAGGCCAG<br>ACACCACCATGGACATTC<br>TTTTGTTGACCCTTT
IVS18+1G>T	GRCh38, 17:<br>43063873..<br>43063873	70090	Single<br>substitution<br>G>T	T*-C	Splice donor<br>variant	Worldwide	GGAAAATGGGTAGTTAGC<br>TATTTCTTTAAGTATAATAC<br>TATTTCTCCCCTC	GAGGGGAGAAATAGTATT<br>ATACTTACAGAAATAGCTA<br>ACTACCCATTTTCC

5632T>A	GRCh38, 17: 43045757..43045757	70278	Single substitution T>A	A*-A	Protein change: V1838E	Worldwide	GCACCTGTGGTGACCCGAGAGTGGGAGTTGGACAGTGTAGCACTCTACCAG	CTGGTAGAGTGCTACACTGTCCAACACCCACTCTCGGGTCACCACAGGTGC
300T>G	GRCh38, 17: 43106487.. 43106487	32700	Single substitution T>G	G*-A	Protein change: C61G	African American and European	CAACCAGAAGAAAGGGCCTTCACAGGGTCCTTTATGTAAGAATGATATAAC	GTTATATCATTCTTACATAAAGGACACTGTGAAGGCCCTTTCTTCTGGTTG
5382insC	GRCh38, 17: 43057065.. 43057065	32716	Single duplication	+C	Frameshift variant, non-coding transcript variant	Worldwide	CAAGGTCCAAAGCGAGCAAGAGAAT[C]CCCAGGACAGAAAGGTAAAGCTCCC	GGGAGCTTTACCTTTCTGTCCTGGGATTCTCTTGCTCGCTTTGGACCTTG
2594delC	GRCh38, 17: 43093056.. 43093056	46028	Single deletion	−C	Frameshift variant, intron variant, non-coding transcript variant	Originated in Central Europe and most common alterations in Northern Europe	GTTGTTCCAAAGATAATAGAAATGA~~C~~ACAGAAGGCTTTAAGTATCCATTGG	CCAATGGATACTTAAAGCCTTCTGTGTCATTTCTATTATCTTTGGAACAAC

[a]BIC (Breast Cancer Information Core): http://research.nhgri.nih.gov/bic/. Nucleotide number according to GenBank U14680.1. http://www.ncbi.nlm.nih.gov/entrez/viewer.fcgi?db=nucleotide&val=555931
[b]http://www.ensembl.org
[c]Twenty five nucleotides before and after the mutation point (letters in black). The mutant nucleotide is marked in red.
[d]Oligonucleotides are 5'-modified with a thiol group.

Supplementary Table 3. Assigned names and information of single stranded DNA

Assigned name	Sequence (5'→3')	5'-modification
ssDNA-1	GCAGTAACGCTATGTGACCGAGAAGGATTCGCATTTG TAGTCTTGAGCCCGCACGAAACCTGGACACCCCTAAG CAACTCCGTACCAGATGGGAACAGCA	Thiol
ssDNA-2	TGCTGTTCCCATCTGGTACGGAGTTGCTTAGGGGTGT CCAGGTTTCGTGCGGGCTCAAGACTACAAATGCGAAT CCTTCTCGGTCACATAGCGTTACTGC	Thiol
homoDNA	ATTGAAAGTTGCAGAATCTGCCCAGAGTCCAGCTGCT GCTCATACTACTGA	None
New 64-bp probe	GAAGCCATTGTCCTCTGTCCAGGCATCTGGCTGCACA ACCACAATTGGGTGGACACCCTGGATC	Thiol

Supplementary Table 4. Comparison of currently used methods for detection of single point mutations

Method[a]	Artifacts from labels and method	DNA volume & concentra -tion for typical detection	Protein concentra -tion required	Limit of detection	Accuracy[b]	Potential for recognizing specific types of mutation	Detection time	Potential for automation with standardization	Feasibility for biomedical use
SPR[6-8]	Label-free with dynamic monitoring; SSB must be used to prevent non-specific surface binding.	100 µl of 1 mM	100 nM	100 nM	< 50% without SSB, ~80% with SSB (causes signal loss).	No[c]	15 min	Limited. Automated on BIACORE, but MutS continues to bind to PCR products without mismatches, limiting clinical applications.	Possible to fabricate a chip (bulk detection).
ECA[9-11]	Label-free without dynamic monitoring; MutS must be immobilized on electrode while electric field disrupts the original MutS-DNA interaction.	10 ml of 2 µM	150 µg/ml	44 nM	Difficult to eliminate non-specific binding.	No	3 h	No. Immobilization of unquantifiable MutS hinders standardization. The electrode and measuring solution need refreshing, hindering automation.	Possible to fabricate a kit with required measuring solution (caution: toxicity due to $[Fe(CN)_6]^{3-}$.
QCM[12,13]	Label-free without dynamic monitoring; requires special surface treatments and ultra-clean and stable apparatus and analytical platform.	50 µl of 5 µM	100 nM	1 nM	Not mentioned. Discrimination ratio between MM and PM DNA is 2.9–5.0.	No	30 min	Limited. Standardized by PzTools, but QCM frequency and damping parameters must be adjusted in different batches of measurements.	Practical application is difficult.

AFM[14,15]	Label-free without dynamic monitoring; DNA needs to be treated with spin-stretcher. Detection requires glutaraldehyde to image multiprotein complexes on DNA.	400 ng; large-sized DNA (~500 bps) is required	125 nM	NA	Not mentioned. In a typical 2 × 2 μm field of view, roughly 10% of DNA is bound by MutS.	No	1 h	No. Optimization is necessary each time to determine the shortest cross-linking time and least amount of cross-linking agent required, while area of interest cannot be standardized.	Ineffective for biomedical applications.
smFRET[16-20]	Label-free with dynamic monitoring; require large amounts of reagent and many processing steps (e.g., MutS requires fluorophore modification at a pre-concentration > 3 μM in buffers to achieve a labeling efficiency of < 55%).	1 ml of 150 nM	> 3 μM in modificati on; 300 nM in detection	10 nM	~70% after 3 rounds of separation (causes signal loss)	Yes, but physiologic al conditions are disrupted for native MutS-protein interactions.	15 min	No. MutS is modified with fluorophores and tends to form dimers.	Possible to fabricate a kit if fluorescentl y labeled MutS can be stably produced at a reasonable cost.
sNPS (this work)	Label-free with dynamic monitoring; detection conditions (e.g. signal generator) preserve the physiological conditions for MutS-protein interactions, without requiring additional chemicals.	30 μl of 50 nM	6.17 nM (able to recognize 4 MutS)	8.63 nM (around five DNA strands)	Almost 100% based on single-particle microfluidic platform	Yes	15 min	Automated by Warner Instruments and Marzhauser Sensotech; particles and protocols can be standardized.	Possible to fabricate a chip (lab-on-a-chip).

[a]Abbreviations: AFM, atomic force microscopy; ECA, electrochemical array; MM, mismatched; NA, not applicable; PM, perfectly matched; QCM, quartz crystal microbalance; smFRET, single-molecule fluorescence resonance energy transfer; sNPS, single NP sensing; SPR, surface plasmon resonance; SSB, single-strand binding protein.
[b]Efficiency of eliminating non-specific MutS binding.
[c]The method can discriminate between a mismatched double-stranded DNA and a perfectly matched one; however, it cannot distinguish between specific types of mismatch; e.g., signals for CC, AC, and GG mutations overlap.

Supplementary Note 1. Summary and comparison of currently used methods for detection of single-base mutations

Point mutations are the most difficult genetic alteration to detect due to their subtle nature. The methods currently used to detect point mutations are summarized in Supplementary Fig. 14.

In the first set of methods (Assay with PCR), mutations are analyzed after the target sequence has been amplified by PCR, which is labor-intensive and consumes large amounts of reagent. Methods including sequencing, gel mobility shift assay, and filter/chip binding assays require the labeling of probes, which can introduce artifacts; in addition, they require samples with a high concentration (e.g., DNA at a concentration of 1 mM or 15 fmol) for visualization, and thus have low resolution and sensitivity. Owing to these disadvantages, we have not compared our system to these methods. However, we emphasize that PCR is considered as the gold standard and remains the definitive approach for studying basic biological mechanisms of diseases, which are the basis of application-based medicine and biosensors.

In the second set of methods (Assay without PCR), mutations are directly analyzed after binding of a mismatch repair (MMR) protein. These approaches meet the requirements of advanced sensors through rapid, direct, and sensitive readout without prior knowledge of sequences. In fact, there are many methods that detect the presence of single base changes by quantifying protein-binding affinity; however, none of these takes into account the influence

of the in vitro environment on the potential of point mutations to be recognized by the MMR protein. The existing methods are compared in the Supplementary Table 4.

Especially for nanoplasmonic sensors, the techniques can be divided into two types: techniques based on surface plasmon resonance (SPR) that use gold-coated substrate for bulk measurements[6-8]; and a technique developed by our research group based on localized (L)SPR that uses single nanoparticles (sNPs) for molecular detection[21]. SPR sensors can detect the presence of mutations but cannot distinguish between different types of point mutation. As in the case of most other sensing techniques consisting of planar surfaces, SPR sensors have the following limitations: planar surfaces have difficulty in localizing and separating sensing elements; there is non-specific surface binding of molecules (e.g., MutS binds to DNA in which there are no mismatches); extraneous molecules (e.g., single-strand binding protein [SSB]) are required to prevent non-specific binding and signal disturbance, resulting in a 10 times higher limit of detection (LOD) and 20% lower accuracy than our sNP sensing (sNPS) method; and MutS must be concentrated to 100 nM (10 times higher than in our system) for effective signal generation. In order to establish an atlas of protein-DNA binding affinities, a sensor must preserve intrinsic biomolecular interactions while rapidly detecting and differentiating between subtle variations. Our sNPS system meets these criteria, as detailed in the following paragraphs.

In terms of accuracy, our sensor monitored protein-DNA interactions without disturbance from additional artifacts or signal sources. The gold bridged NPs transmit low-energy white light, which preserves the intrinsic MutS-DNA interactions and thereby

identifies distinct types of point mutation. In contrast, exogenous molecules for optimizing detection (i.e., SSB protein for SPR, DNA spin-stretcher for atomic force microscopy [AFM], and labels for single-molecule fluorescence resonance energy transfer [smFRET]) alter biomolecular conditions, while high-energy light (e.g., laser in some SPR methods) and electric fields (employed in electrochemical array [ECA] and quartz crystal microbalance [QCM]) disrupt MutS-DNA interactions. It should be noted that the surface charge effect is essential for MutS recognition of point mutations.

For enhanced sensitivity, we originally fabricated a 51-bp probe that precisely fit the MutS footprint. MutS-DNA binding drastically altered plasmonic resonance, resulting in the detection of as few as five MutS binding events, indicating molecular-level sensitivity. Only QCM has comparable sensing capacity; however, it cannot exclude non-specific binding and requires an ultra-clean and stable apparatus and analytical platform, which limits its practical application in biomedical assay.

To optimize sampling, we designed gold-bridged NPs with two spherical ends and a nanobridge between the two ends. The uniquely small sensing area required only 30 μl of sample solution for one round of detection. Importantly, 10 nM MutS was sufficient for effective signal generation. The requirements of a small sample volume and small amount of signal generator are essential for advanced sensors. The smFRET method has an LOD (~10 nM) that is similar to that of our system; however, it requires > 1000 μl sample for detection using 300 nM MutS; and the MutS requires fluorophore modification at a pre-concentration >

3 μM in buffers to achieve a labeling efficiency of < 55%. Moreover, the modification changes the behavior of MutS (e.g. favors dimerization).

For processing, we combined sNPS with a microfluidic chamber. Most biomolecules flowing through the detection channel pass within the plasmonic field of the single particle, where targets encountered nanoprobes while non-targets were washed away (total detection time was 15 min, and background noises from instrumental, analytical and nanoparticle factors and nonspecific bindings were lower than 0.700 nm). Thus, our method achieved parallel readout with high throughput and accuracy, and was operationally simplified since it did not require separation, concentration, or localization of sensing elements. Bulk measurements by SPR, smFRET, ECA, and QCM involve complex processing steps to separate and concentrate analytes. For example, the detection accuracy of smFRET is reduced to 70% as a result of three analyte purification steps, whereas in AFM an area of interest must be selected that cannot be standardized for quantitative analysis.

Ultimately, our sensor reported the presence and number of DNA mutations and differentiated between individual point mutations that could be classified into four types based on their potential for recognition by MutS. This work demonstrated an atlas of MutS affinities for different point mutations based on recognition of a countable number of biomolecular binding events.

Supplementary Note 2. Interpretation of different potentials of point mutations to be recognized by MutS (GT > GG > +C > AA > TC > −C > AC > GA)

Global structural changes are typically greater in transversion mismatches (green circles in Fig. 5) than in transitions (blue circles). However, the order determined in the present study was unrelated to mutation category (i.e., transversion or transition). In fact, recent studies have shown that single point mutations only moderately impact global duplex structure[22]. Transversions and transitions do not dramatically modify the helix, and the structure of DNA is sufficiently plastic to accommodate single mismatches.

MutS protein specifically recognizes single-base substitutions as well as insertion/deletion mismatches (I/D) of 1–3 nucleotides[23]. One MutS uses its conserved Phe-Xaa-Glu motif to bind to one mutant site in a common binding mode[1], by which the phenylalanine, Phe 36, is to be stacking on one of the mismatched bases. The same base is also seen as forming a hydrogen bond with the glutamate, Glu 38. This hydrogen bond involves the N7 if the base stacking on Phe 36 is a purine and the N3 if it is a pyrimidine. The recognition of a given mutation by MutS is determined by the geometry and dynamics of mismatched bases around the glycosidic bond (Supplementary Fig. 15). Our results indicate that the GT–MutS complex had the most highly ordered geometry because the mismatched G adopted a conformation similar to that of the cognate base at this position; consequently, the positioning of the 3' hydroxyl and binding sites remained mostly intact, with G forming an H bond with Asp but perturbing the orientation of the adjacent G to form an additional H bond with Glu. The remaining base (T) also formed an H bond with Glu while stacking with Phe.

These three H bonds and stacking forces allowed the protein to easily wedge into the widened minor groove of DNA. It was recently shown that the ion cloud around the GT transition is similar to that observed in canonical pairing[22]. In contrast, AC—the other transition mutation—adopted a protonated conformation that caused too great a distortion of DNA to allow identification of mismatch location, and was unable to form more than a single H bond in the dominant tautomeric state[24,25].

Unlike transitions, transversions produce twisted conformations. With the exception of GA mismatches, purine-purine transversions induce greater rotation and translation of one base with respect to the other than do pyrimidine-pyrimidine transversions[22]. Such movement allows bases to engage in more interactions with MutS and explains why GG and AA ranked higher than TC. The GA transversion has an under-twisted conformation, and the bases are precluded from interactions with the protein[24], resulting in a low detection frequency.

Mismatched bases engage in promiscuous dynamic H bonding on a nanosecond time scale. This so-called breathing generates significant changes in base orientation around glycosidic bonds, leading to highly efficient binding between nucleic acid and protein. The breathing profile of GG showed a longer time evolution and higher total breathing percentage than that of AA, consistent with their detection ranks. Additionally, GA (which has a low breathing frequency)[22] was the least-recognized purine-purine mutation.

Interestingly, MutS showed a lower binding affinity for DNA with bulging mutations (red circles in Fig. 5) than for DNA with base substitutions (i.e., GG > +C, TC > −C). MutS preferentially recognizes and repairs intrahelical over extrahelical bases[26,27]. Although it

remains unclear whether it forms an extra loop or is accommodated within the helix, the bulging mutation results in inadequate alterations in base orientation towards the Phe-Xaa-Glu motif of MutS. Notably, MutS is better at recognizing +C than −C, since a flipped-out pyrimidine provides more opportunities for specific interactions with conserved Phe or Glu residues[1].

Supplementary References

1. Natrajan, G., *et al.* Structures of *Escherichia coli* DNA mismatch repair enzyme MutS in complex with different mismatches: a common recognition mode for diverse substrates. *Nucleic Acids Res* **31,** 4814-4821 (2003).
2. Lamers, M. H., *et al.* The crystal structure of DNA mismatch repair protein MutS binding to a G·T mismatch. *Nature* **407,** 711-717 (2000).
3. Skelly, J. V., Edwards, K. J., Jenkins, T. C. & Neidle, S. Crystal-structure of an oligonucleotide duplex containing G·G base-pairs: influence of mispairing on DNA backbone conformation. *Proc Natl Acad Sci USA* **90,** 804-808 (1993).
4. Obmolova, G., Ban, C., Hsieh, P. & Yang, W. Crystal structures of mismatch repair protein MutS and its complex with a substrate DNA. *Nature* **407,** 703-710 (2000).
5. Hunter, W. N., Brown, T. & Kennard, O. Structural features and hydration of a dodecamer duplex containing two C.A mispairs. *Nucleic Acids Res* **15,** 6589-6606 (1987).
6. Gotoh, M., *et al.* Rapid method for detection of point mutations using mismatch binding protein (MutS) and an optical biosensor. *Genet Anal-Biomol E* **14,** 47-50 (1997).
7. Margara, L. M., Fernandez, M. M., Malchiodi, E. L., Argarana, C. E. & Monti, M. R. MutS regulates access of the error-prone DNA polymerase Pol IV to replication sites: a novel mechanism for maintaining replication fidelity. *Nucleic Acids Res* **44,** 7700-7713 (2016).
8. Groothuizen, F. S., *et al.* Using stable MutS dimers and tetramers to quantitatively analyze DNA mismatch recognition and sliding clamp formation. *Nucleic Acids Res* **41,** 8166-8181 (2013).
9. Cho, M., *et al.* Electrochemical detection of mismatched DNA using a MutS probe. *Nucleic Acids Res* **34,** e75 (2006).
10. Chen, H., *et al.* Electrochemical scanning of DNA point mutations via MutS protein-mediated mismatch recognition. *Biosensors & Bioelectronics* **24,** 1955-1961 (2009).
11. Kim, S., *et al.* Recognition of Single Mismatched DNA Using MutS-Immobilized Carbon Nanotube Field Effect Transistor Devices. *J Phys Chem B* **113,** 12164-12168 (2009).
12. Su, X. D., Robelek, R., Wu, Y. J., Wang, G. Y. & Knoll, W. Detection of point mutation and insertion mutations in DNA using a quartz crystal microbalance and MutS, a mismatch binding protein. *Anal Chem* **76,** 489-494 (2004).

13. Su, X. D., Wu, Y. J., Robelek, R. & Knoll, W. Surface plasmon resonance spectroscopy and quartz crystal microbalance study of MutS binding with single Thymine-Guanine mismatched DNA. *Front Biosci* **10,** 268-274 (2005).

14. LeBlanc, S., *et al.* Using Atomic Force Microscopy to Characterize the Conformational Properties of Proteins and Protein-DNA Complexes That Carry Out DNA Repair. *Method Enzymol* **592,** 187-212 (2017).

15. Sun, H. B. & Yokota, H. MutS-mediated detection of DNA mismatches using atomic force microscopy. *Anal Chem* **72,** 3138-3141 (2000).

16. Weninger, K. Dynamics of DNA Mismatch Repair Revealed by Single Molecule FRET. *Biophys J* **102,** 235a-235a (2012).

17. Lang, W. H., *et al.* Conformational trapping of Mismatch Recognition Complex MSH2/MSH3 on repair-resistant DNA loops. *Proc Natl Acad Sci USA* **108,** E837-E844 (2011).

18. Jeong, C., *et al.* MutS switches between two fundamentally distinct clamps during mismatch repair. *Nat Struct Mol Biol* **18,** 379-385 (2011).

19. Qiu, R. Y., *et al.* MutL traps MutS at a DNA mismatch. *Proc Natl Acad Sci USA* **112,** 10914-10919 (2015).

20. Cristovao, M., *et al.* Single-molecule multiparameter fluorescence spectroscopy reveals directional MutS binding to mismatched bases in DNA. *Nucleic Acids Res* **40,** 5448-5464 (2012).

21. Ma, X., Truong, P. L., Anh, N. H. & Sim, S. J. Single gold nanoplasmonic sensor for clinical cancer diagnosis based on specific interaction between nucleic acids and protein. *Biosens Bioelectron* **67,** 59-65 (2015).

22. Rossetti, G., *et al.* The structural impact of DNA mismatches. *Nucleic Acids Res* **43,** 4309-4321 (2015).

23. Schofield, M. J., *et al.* The Phe-X-Glu DNA binding motif of MutS - The role of hydrogen bonding in mismatch recognition. *J Biol Chem* **276,** 45505-45508 (2001).

24. Johnson, S. J. & Beese, L. S. Structures of mismatch replication errors observed in a DNA polymerase. *Cell* **116,** 803-816 (2004).

25. Brown, J., Brown, T. & Fox, K. R. Affinity of mismatch-binding protein MutS for heteroduplexes containing different mismatches. *Biochem J* **354,** 627-633 (2001).

26. Romanova, N. V. & Crouse, G. F. Different roles of eukaryotic Muts and Mutl complexes in repair of small insertion and deletion loops in yeast. *Plos Genet* **9,** e1003920 (2013).

27. Fazakerley, G. V., *et al.* Structures of mismatched base-pairs in DNA and their recognition by the *Escherichia coli* mismatch repair system. *EMBO J* **5,** 3697-3703 (1986).
