# Supplementary material for: Single gold-bridged nanoprobes for identification of single point DNA mutations
Source: Nat Commun. 2019 Feb 19;10:836. doi: 10.1038/s41467-019-08769-y (PMC6381086; doi:10.1038/s41467-019-08769-y)
Supplement: Supplementary file 2 — Reporting Summary [file 41467_2019_8769_MOESM2_ESM.pdf]

nature research

Corresponding author(s): Sang Jun Sim
Last updated by author(s): Jan 20, 2019

Reporting Summary

Nature Research wishes to improve the reproducibility of the work that we publish. This form provides structure for consistency and transparency in reporting. For further information on Nature Research policies, see Authors & Referees and the Editorial Policy Checklist.

Statistics

For all statistical analyses, confirm that the following items are present in the figure legend, table legend, main text, or Methods section.

n/a	Confirmed	
☐	☒	The exact sample size (*n*) for each experimental group/condition, given as a discrete number and unit of measurement
☐	☒	A statement on whether measurements were taken from distinct samples or whether the same sample was measured repeatedly
☐	☒	The statistical test(s) used AND whether they are one- or two-sided<br>*Only common tests should be described solely by name; describe more complex techniques in the Methods section.*
☐	☒	A description of all covariates tested
☐	☒	A description of any assumptions or corrections, such as tests of normality and adjustment for multiple comparisons
☐	☒	A full description of the statistical parameters including central tendency (e.g. means) or other basic estimates (e.g. regression coefficient) AND variation (e.g. standard deviation) or associated estimates of uncertainty (e.g. confidence intervals)
☐	☒	For null hypothesis testing, the test statistic (e.g. *F*, *t*, *r*) with confidence intervals, effect sizes, degrees of freedom and *P* value noted<br>*Give P values as exact values whenever suitable.*
☒	☐	For Bayesian analysis, information on the choice of priors and Markov chain Monte Carlo settings
☒	☐	For hierarchical and complex designs, identification of the appropriate level for tests and full reporting of outcomes
☒	☐	Estimates of effect sizes (e.g. Cohen's *d*, Pearson's *r*), indicating how they were calculated

Our web collection on statistics for biologists contains articles on many of the points above.

Software and code

Policy information about availability of computer code

Data collection	The commercial software tool COMSOL 5.3a was used to produce all optical simulation data. The software ImageJ was used to measure sizes of nanostructures. The commercial software DigitalMicrograph was used to analyse FFT patterns. The WinSpec software was used to perform imaging and spectroanalysis of single nanoparticles. The software GENETYX 4.0 was used to compute fragmentation maps of genes after restriction digestion.
Data analysis	The data including electric and magnetic fields from COMSOL, FFT image from DigitalMicrograph, CCD and true-colour images from WinSpec, and DNA fragmentation maps from GENETYX were directly used. The data of frequencies of the plasmon resonance from COMSOL and XRD data were represented by Excel. All spectral data were analysed using the Origin2018 software.

For manuscripts utilizing custom algorithms or software that are central to the research but not yet described in published literature, software must be made available to editors/reviewers. We strongly encourage code deposition in a community repository (e.g. GitHub). See the Nature Research guidelines for submitting code & software for further information.

Data

Policy information about availability of data

All manuscripts must include a data availability statement. This statement should provide the following information, where applicable:

- Accession codes, unique identifiers, or web links for publicly available datasets
- A list of figures that have associated raw data
- A description of any restrictions on data availability

The data supporting the findings of this study are available within the article and its Supplementary Information. The source data underlying all graphs in Figs. 1b, d, 2b, c, e, f and 3–7, and Supplementary Figs. 4e, 5 and 10 are provided as a Source Data file. All data are available from the corresponding author upon reasonable request. A reporting summary for this Article is available as a Supplementary Information file.

Field-specific reporting

Please select the one below that is the best fit for your research. If you are not sure, read the appropriate sections before making your selection.

☒ Life sciences ☐ Behavioural & social sciences ☐ Ecological, evolutionary & environmental sciences

For a reference copy of the document with all sections, see nature.com/documents/nr-reporting-summary-flat.pdf

Life sciences study design

All studies must disclose on these points even when the disclosure is negative.

Sample size	No sample size calculation was performed. Since the spectrum of BRCA1 mutations in different carrier populations is broad, the single nanoparticle sensing system in this study has been designed to detect eight most common and important point mutations based on literature researches.
Data exclusions	No data were excluded from the analyses.
Replication	Three parallel tests in all the studies were successful.
Randomization	Individual nanoparticles were randomly analysed. The overall performance of the single nanoparticle sensing system was confirmed to be stable and reliable.
Blinding	Three cell lines were blinding for the proof-of-principle demonstration of the method developed in this study.

Reporting for specific materials, systems and methods

We require information from authors about some types of materials, experimental systems and methods used in many studies. Here, indicate whether each material, system or method listed is relevant to your study. If you are not sure if a list item applies to your research, read the appropriate section before selecting a response.

Materials & experimental systems

n/a	Involved in the study	
☒	☐	Antibodies
☐	☒	Eukaryotic cell lines
☒	☐	Palaeontology
☒	☐	Animals and other organisms
☒	☐	Human research participants
☒	☐	Clinical data

Methods

n/a	Involved in the study	
☒	☐	ChIP-seq
☒	☐	Flow cytometry
☒	☐	MRI-based neuroimaging

Eukaryotic cell lines

Policy information about cell lines

Cell line source(s)	We prepared biological DNA samples from the human cancer cell lines HCC1937 (ATCC, CRL-2336), SUN251 (Korean Cell Line Bank, 00251) and MCF7 (ATCC, HTB-22).
Authentication	The authentication of HCC1937 has been reported in Cancer Research 58, 3237-3242, 1998. And the authentication of SNU251 has been reported in Experimental & Molecular Medicine, 47, e195, 2015.
Mycoplasma contamination	No mycoplasma contamination.
Commonly misidentified lines (See ICLAC register)	None.
